# Effects from medications on functional biomarkers of aging in three longitudinal studies of aging in Sweden

**DOI:** 10.1111/acel.14132

**Published:** 2024-03-01

**Authors:** Thaís Lopes De Oliveira, Bowen Tang, Ge Bai, Arvid Sjölander, Juulia Jylhävä, Deborah Finkel, Nancy L. Pedersen, Linda B. Hassing, Chandra A. Reynolds, Ida K. Karlsson, Sara Hägg

**Affiliations:** ^1^ Department of Medical Epidemiology and Biostatistics Karolinska Institutet Stockholm Sweden; ^2^ Department of Neurobiology, Care Sciences and Society, Aging Research Center Karolinska Institutet Stockholm Sweden; ^3^ Department of Women's and Children's Health Uppsala Universitet Uppsala Sweden; ^4^ Faculty of Social Sciences (Health Sciences) and Gerontology Research Center (GEREC) University of Tampere Tampere Finland; ^5^ School of Health and Welfare, Institute of Gerontology Jönköping University Jönköping Sweden; ^6^ Center for Economic and Social Research University of Southern California Los Angeles California USA; ^7^ Department of Psychology University of Gothenburg Gothenburg Sweden; ^8^ Centre for Ageing and Health University of Gothenburg Gothenburg Sweden; ^9^ Department of Psychology The University of California at Riverside Riverside California USA; ^10^ Department of Psychology and Neuroscience Institute for Behavioral Genetics, University of Colorado Boulder Boulder Colorado USA

**Keywords:** aging, cognitive function, frailty, medication, physical function

## Abstract

Antihypertensive, lipid‐lowering, and blood glucose‐lowering drugs have slowed down the aging process in animal models. In humans, studies are limited, have short follow‐up times, and show mixed results. Therefore, this study aimed to estimate the effects of commonly used medications on functional aging, cognitive function, and frailty. We included information on individuals from three Swedish longitudinal population‐based studies collected between 1986 and 2014. Our exposures were the 21 most used groups of medications among individuals aged 65 years and older in the Swedish population in 2022. Functional aging index (*n* = 1191), cognitive function (*n* = 1094), and frailty index (*n* = 1361) were the outcomes of interest. To estimate the medication effects, we used a self‐controlled analysis, where each individual is his/her own control, thereby adjusting for all time‐stable confounders. The analysis was additionally adjusted for time‐varying confounders (chronological age, Charlson Comorbidity Index, smoking, body mass index, and the number of drugs). The participants were 65.5–82.8 years at the first in‐person assessment. Adrenergics/inhalants (effect size = 0.089) and lipid‐modifying agents/plain (effect size = 0.082) were associated with higher values of cognitive function (improvement), and selective calcium channel blockers with mainly vascular effects (effect size = −0.129) were associated with lower values of the functional aging index (improvement). No beneficial effects were found on the frailty index. Adrenergics/inhalants, lipid‐modifying agents/plain, and selective calcium channel blockers with mainly vascular effects may benefit functional biomarkers of aging. More research is needed to investigate their clinical value in preventing adverse aging outcomes.

## Graphical abstract

Adrenergics/inhalants, lipid‐modifying agents, and selective calcium channel blockers with mainly vascular effects may benefit functional biomarkers of aging (functional aging index, cognitive function, and frailty index). Their effects could be due to positive pleiotropic effects related to better cellular and physiological performance.

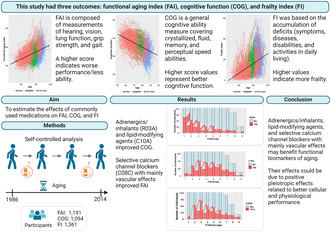

**Abbreviations**
A02B: Drugs for peptic ulcer and gastro‐oesophageal reflux disease
A06A: Drugs for constipation
A10B: Blood glucose lowering drugs/excl. Insulins
A12A: Calcium
ATC: Anatomical Therapeutic Chemical Classification System
B01A: Antithrombotic agents
B03B: Vitamin B12 and folic acid
BMI: Body mass index
C03C: High‐ceiling diuretics
C07A: Beta‐blocking agents
C08C: Selective calcium channel blockers with mainly vascular effects (dihydropyridines)
C09A: Ace inhibitors/plain
C09C: Angiotensin ii receptor blockers arbs/plain
C10A: Lipid‐modifying agent/plain
CCB: Calcium channel blockers
CCI: Charlson comorbidity index
cGEE: Conditional generalized estimating equation models
COG: Cognitive function
D02A: Emollients and protectives
FAI: Functional aging index
FI: Frailty index
G04B: Urologicas
GENDER: Longitudinal Study of Gender Differences in Health Behavior and Health among Elderly
H03A: Thyroid preparations
IPT: In‐person testing
M01A: Anti‐inflammatory and antirheumatic products/non‐steroids
MAR: Missing at random
MCAR: Missing completely at random
N02A: Opioids
N02B: Other analgesics and antipyretics
N05C: Hypnotics and sedatives
N06A: Antidepressants
NEAR: National E‐infrastructure for Aging Research
OCTO‐Twin: Origins of Variance in the Oldest‐Old: Octogenarian Twins
PCA: Principal components analysis
R03A: Adrenergics/inhalants.
SATSA: Swedish Adoption/Twin Study of Aging

## 1 INTRODUCTION

Biological aging is a process involving cellular, molecular, physiological, and functional changes during life (López‐Otín et al., 2023). Over the years, biomarkers of aging have emerged to better describe the biological aging process and to complement the risk prediction of chronological age on health outcomes (Jylhävä et al., 2017).

Functional biomarkers of aging include, for example, walking speed, grip strength, muscle mass, frailty (a syndrome of deficits that combines physical and/or psychological conditions), and cognitive function. The aging process is often associated with the worsening of these biomarkers and the development of multimorbidity (Forslund et al., 2021; Manfredi et al., 2019). Cognitive impairment (loss of cognitive function) and frailty have a high prevalence in Europe (Manfredi et al., 2019; Pais et al., 2020), and their deterioration increases the care costs to society, individual vulnerability, dependency, risk of diseases, and mortality (Bai et al., 2021; Lenox‐Smith et al., 2018; Li et al., 2020; Pushpakom et al., 2018). Considering these burdens, cost‐effective approaches to identify treatments that could improve functional biological aging are relevant. One strategy is to identify new medical uses for approved medications, also known as drug repurposing (Pushpakom et al., 2018).

In animal models, compounds from drugs for cardiovascular, diabetes, and urinary disorders have been related to anti‐aging properties with potential beneficial effects outside of their original indication (Barardo et al., 2017). So far, in humans, the effects of these drugs on functional biomarkers of aging are inconclusive (DeLoach & Beall, 2018; Espinoza et al., 2019; Zhu et al., 2018). Among oral anti‐diabetic drugs, metformin improved cognitive function in patients with diabetes (Teng et al., 2021; Zhang, Li, et al., 2020). For lipid‐lowering and antihypertensive drugs, simvastatin, atorvastatin, beta‐blockers, dihydropyridine calcium channel blockers, and potassium‐sparing diuretics are associated with reductions in dementia and Alzheimer's risk (DeLoach & Beall, 2018; Zhu et al., 2018).

Although benefits on biological aging are reported in observational studies and randomized clinical trials, these studies have limitations concerning confounding by indication, disease duration, or limited follow‐up time. Furthermore, the effects of drugs commonly used by the older Swedish population (e.g., medicines for thyroid, respiratory, rheumatic, inflammatory, and mental disorders) on functional biomarkers of aging are still unknown (Socialstyrelsen, 2022). Hence, evidence is still needed to support the use of aforementioned drugs to mitigate adverse aging outcomes. Our study aims to investigate the effects of different pharmacological subgroups on functional aging, cognitive function, and frailty.

## 2 METHODS

### 2.1 Study population

We used harmonized information from three longitudinal studies; the Swedish Adoption/Twin Study of Aging (SATSA), a Longitudinal Study of Gender Differences in Health Behavior and Health among Elderly (GENDER), and the Origins of Variance in the Oldest‐Old: Octogenarian Twins (OCTO‐Twin). SATSA and OCTO‐Twin are same‐sex twin studies, while GENDER is an opposite‐sex twin study (Gold et al., 2002; McClearn et al., 1997; Pedersen et al., 1991). All of them are part of the National E‐infrastructure for Aging Research (NEAR). The NEAR project coordinates databases of several longitudinal studies of aging in Sweden and is a collaboration between eight Swedish universities (NEAR, 2022).

We analyzed data collected in extensive in‐person testing (IPT). Each IPT included a cognitive function battery, physical tests, and health information. We included up to nine, three, and five IPTs for SATSA, GENDER, and OCTO‐Twin, respectively (Figure S1; Gold et al., 2002; McClearn et al., 1997; Pedersen et al., 1991). Only complete individual data was included in the analyses (Table S1). We excluded participants if they did not report any medication names but said yes for using any medication; and if they had only one IPT. We also excluded missing values in outcome or time‐varying confounders at baseline or at any longitudinal follow‐up; however, if the participants had at least two repeated measurements for all variables at any moment during the study, they were included (Figure S2). For example, participants were included if they had measurements in IPT 3 and 5, but without baseline information.

### 2.2 Exposure variables

The exposures were the 20 most common medications taken by the Swedish population aged 65 years or more in 2022. We selected these medications from the Statistics Sweden database, which contains information on medicines collected at pharmacies through prescriptions between 2006 and 2022 (Socialstyrelsen, 2022). The supporting information file has additional information on our medication selection (Tables S2 and S3). The drugs in SATSA, GENDER, and OCTO‐Twin were self‐reported. For SATSA, the self‐reporting data was validated with information from the Swedish Prescribed Drug Register by Tang et al. (2023). They found that “98–100% of the SATSA participants who reported using the drugs purchased the corresponding drugs within 1 year before the IPT data, while 88–99% of the participants who did not report using the drugs did not purchase the drugs” (Tang et al., 2023).

We classified the drugs according to the third level (pharmacological subgroup) of the Anatomical Therapeutic Chemical Classification System (ATC), which is a system that divides active substances into groups according to their properties (WHO, 2022). The choice of the third ATC level relies on including more detailed information about medications. For example, A10—drugs used in diabetes (second level) does not detail insulin use. In the third level, this information is available (A10B—Blood glucose lowering drugs/excl insulins). The medication groups were coded as dummy variables (0 = no medication use vs. 1 = medication use) in each ITPs according to the ATC codes described below.

1. A02B: Drugs for peptic ulcer and gastro‐esophageal reflux disease
2. A06A: Drugs for constipation
3. A10B: Blood glucose lowering drugs/excl. Insulins
4. A12A: Calcium
5. B01A: Antithrombotic agents
6. B03B: Vitamin B12 and folic acid
7. C03C: High‐ceiling diuretics
8. C07A: Beta‐blocking agents
9. C08C: Selective calcium channel blockers with mainly vascular effects (dihydropyridines)
10. C09A: Ace inhibitors/plain (it does not include combinations of ace inhibitors and diuretics, calcium channel blockers, or beta‐blocking agents).
11. C09C: Angiotensin ii receptor blockers (arbs)/plain
12. C10A: Lipid‐modifying agent/plain
13. D02A: Emollients and protectives
14. G04B: Urologicals
15. H03A: Thyroid preparations
16. M01A: Anti‐inflammatory and antirheumatic products/nonsteroids
17. N02A: Opioids
18. N02B: Other analgesics and antipyretics
19. N05C: Hypnotics and sedatives
20. N06A: Antidepressants
21. R03A: Adrenergics/inhalants.

Individuals with variation in medicine intake over the IPTs (e.g., using the medication in IPT 1 and IPT 3 but not using it in ITP 2) contributed directly to the estimated medication effects in the self‐controlled analysis. Individuals with no variation in medication intake (never or always used the drug) contributed indirectly to the estimated medication effects by contributing to the estimated effects of the time‐varying confounders. The medicine intake variation is represented in Figure S3A,B.

### 2.3 Outcome variables

This study had three outcomes: functional aging index, cognitive function, and frailty index. The functional aging index (FAI) is a measure based on biomarkers of aging that quantify functioning, for example, lung function. The index was created using factor analysis, and the biomarker validation is based on five criteria which were as follows: “(a) candidate component measures must be associated with (functional) health status; (b) functioning on the measures should generally decline with age; (c) declines in functioning should not saturate too early in the aging process; (d) measures should tap a range of domains; and (e) in longitudinal studies, component measures must be consistent over measurement waves” (Finkel et al., 2019). The FAI items were sensory functioning, lung function, gait, and grip strength (Table S4). Sensory functioning was composed of self‐reported hearing and self‐reported vision. The lung function was the peak expiratory flow divided by height in meters‐squared, and gait was the time to walk three meters and return. The grip strength is a measure of hand and forearm muscle strength. It was measured in six attempts (three with each hand) using a dynamometer and vigorimeter. The participant's grip strength is a score (maximum score in kilogram) corrected by sex (Finkel et al., 2019). FAI is a standardized T‐score relative to the sample mean (*M* = 50) and standard deviation (SD = 10) at the baseline. A higher score indicates worse performance/less ability. Details of the construction of FAI can be found elsewhere (Finkel et al., 2019).

The cognitive function (COG) consists of a general cognitive ability measure composed of distinct types of cognitive tests covering crystallized, fluid, memory, and perceptual speed abilities. The measure was derived from principal components analysis (PCA) of four cognitive tests: Synonyms, Block Design, Thurstone's Picture Memory Task, and Symbol Digit. Weights from a first principal component obtained at IPT1 were used to create the COG measure for all IPTs. All COG measure, in all ITP, excluded individuals with dementia. Test scores across IPTs were scaled against their respective IPT1 mean and SD before PCA weights were applied. Afterward, T‐scoring was applied (*M* = 50, SD = 10) on the created component scores (mean‐adjusted by sex). Higher score values represent better cognitive function. Details of the construction of this measure can be found elsewhere (Finkel et al., 2003; Pedersen et al., 1992). The PCA resulted in an individual cognitive composite score comparable between the cohorts, and other studies exemplify the application of this technique across different longitudinal studies (Davies et al., 2018; Li et al., 2020).

The frailty index (FI) was based on the accumulation of deficits approach and was calculated by the sum of the number of deficits a person has, divided by the total number of deficits present in the index (Rockwood & Mitnitski, 2007). Symptoms, diseases, disabilities, and activities in daily living were deficits. SATSA and GENDER had 42 items in the index, while OCTO‐Twin had 41 (Bai et al., 2021). The items included in the FI can be found in the Table S5. The FI is an appropriate measure to be harmonized in distinct studies since it can be composed of similar deficits between cohorts but not necessarily the same. Other studies exemplify its use across cohorts (Bai et al., 2021; Finkel et al., 2019). The FI varies between 0 and 1. Our results present the FI multiplied by 100 to facilitate interpretation, and the estimates represent increments of 1. There were no available FAI and FI measures in IPT1 and IPT4 for SATSA (Figure S1).

### 2.4 Time‐varying confounders

The time‐varying confounders measured in our study were chronological age, Charlson Comorbidity Index (CCI), smoking status, body mass index (BMI), and the number of drugs (according to fifth level ATC codes, since these codes are the chemical substance). All time‐varying confounders were measured in the same date. Age, CCI, BMI, and the number of drugs were continuous variables; smoking status was categorical. The CCI was based on the International Classification of Diseases, from versions 7 to 10. It varied from 0 to 13 (Ludvigsson et al., 2021). Smoking status was treated as 1 = not currently smoking and 2 = currently smoking. BMI was based on self‐reported weight and height. It was calculated in kilograms divided by squared height in meters. The number of drugs varied between 0 and 21.

### 2.5 Statistical analyses

Means, standard deviations, proportions, and frequency were used to describe characteristics of the study population regarding medications and outcomes of interest. Descriptive spaghetti plots of the outcomes over age were presented separately by cohort studies.

The medication effects on FAI, COG, and FI were estimated with a self‐controlled analysis. Technically, we used conditional generalized estimating equation models (cGEE), where each individual entered as a separate stratum in the model. By using each individual as his/her own control this model implicitly adjusts for all time‐stable confounders (Goetgeluk & Vansteelandt, 2008). We used linear models throughout. Thus, the estimated effects (β values) are differences in mean outcomes, comparing “taking the medication” to “not taking the medication”. The cGEE assumptions are independence of the clusters (in our case, individuals), and “the observations within each cluster are conditionally independent given the cluster‐constant covariates”. The supplementary file of Tang et al. has a detailed description of the cGEE model with application in SATSA cohort (Tang et al., 2023). One of the advantages of cGEE is that it obtains more robust estimates with stronger causal evidence. More information on this model can be found elsewhere (Goetgeluk & Vansteelandt, 2008; Zetterqvist et al., 2016).

We fitted two different models:

1. Model 1 refers to estimated crude effects adjusted for age, conducted separately for each medication. Drugs with *p* ≤ 0.15 in model 1 were included in model 2 for further adjustments. Since we are looking for an effect outside of the drug's original indication, we considered *p* ≤ 0.15 to avoid excluding a potentially beneficial medication after adjustments for confounders.
2. Model 2 included all medications from model 1 with *p* ≤ 0.15 and further adjusted for age, CCI, smoking status, BMI, and the number of drugs. Hence, all the selected medications from Model 1 were adjusted for simultaneously.

All models are a bootstrap analysis with resampling of twin pairs 10,000 times. This technique aims to correct the confidence intervals and p‐values for the within twin‐pair correlation. The estimates, in Model 2, with confidence intervals 95% were interpreted as statistically significant. Also, Table S6 shows the p‐values for model 2 estimates considering Bonferroni correction for multiple testing. As a secondary analysis, models were fitted to men and women separately (Tables S7–S9). All analyses were conducted using R software (R Core Team, 2022), version 4.0.5, package tidyverse, and degree.

## 3 RESULTS

Age differed significantly between studies at the first assessed IPT. Individuals in the OCTO‐Twin study were older, had higher FAI, worse cognitive ability, and were more frail. They also had more comorbidities and lower BMI. There were more women in SATSA and OCTO‐Twin studies. Finally, other analgesics and antipyretics (ATC: N02B) were the most used medications in all studies (Table 1 and Table S10).

**TABLE 1:** Descriptives of first assessed study variables for all cohorts (SATSA, GENDER, and OCTO‐Twin).

Variable	SATSA (*n* = 619)	GENDER (*n* = 352)	OCTO‐twin (*n* = 456)	All cohorts (*n* = 1427)
Sex, *n* (%)				
Men	272 (43.9)	178 (50.6)	154 (33.8)	604 (42.3)
Women	347 (56.1)	174 (49.4)	302 (66.2)	823 (57.7)
Functional aging index, *M* (SD)	43.4 (10.8)	42.8 (8.7)	52.2 (10.3)	46.3 (10.8)
Cognitive function, *M* (SD)	55.9 (9.0)	54.7 (7.2)	45.4 (9.3)	53.0 (9.9)
Frailty index, *M* (SD)	8.46 (6.2)	11.5 (6.2)	20.2 (10.1)	14.5 (9.5)
Age (years), *M* (SD)	63.5 (7.8)	74.4 (2.6)	83.2 (2.8)	72.5 (10.1)
Charlson comorbidity index, *M* (SD)	0.12 (0.5)	0.22 (0.7)	0.33 (0.8)	0.21 (0.6)
Body mass index (kg/m^2^), *M* (SD)	25.8 (3.9)	26.6 (3.8)	24.6 (3.7)	25.6 (3.9)
Number of drugs, *M* (SD)	1.74 (1.7)	2.66 (2.3)	3.19 (2.6)	2.43 (2.3)
Smoking, *n* (%)				
Not currently	502 (81.1)	320 (90.9)	416 (91.2)	1238 (86.8)
Currently	117 (18.9)	32 (9.1)	40 (8.8)	189 (13.2)
Ace inhibitors/plain (C09A), *n* yes (%)	11 (1.8)	0 (0)	0 (0)	11 (0.8)
Adrenergics/inhalants (R03A), *n* yes (%)	17 (2.7)	15 (4.3)	20 (4.4)	52 (3.6)
Angiotensin ii receptor blockers (arbs)/plain (C09C), *n* yes (%)	18 (2.9)	0 (0)	0 (0)	18 (1.3)
Antidepressants (N06A), *n* yes (%)	11 (1.8)	11 (3.1)	9 (2.0)	31 (2.2)
Anti‐inflammatory and antirheumatic products/nonsteroids (M01A), *n* (%)	30 (4.8)	41 (11.6)	34 (7.5)	105 (7.4)
Antithrombotic agents (B01A), *n* yes (%)	26 (4.2)	61 (17.3)	17 (3.7)	104 (7.3)
Beta‐blocking agents (C07A), *n* yes (%)	87 (14.1)	57 (16.2)	62 (13.6)	206 (14.4)
Blood glucose lowering drugs/excl. Insulins (A10B), *n* yes (%)	6 (1.0)	15 (4.3)	12 (2.6)	33 (2.3)
Calcium (A12A), *n* yes (%)	8 (1.3)	6 (1.7)	10 (2.2)	24 (1.7)
Drugs for constipation (A06A), *n* yes (%)	11 (1.8)	12 (3.4)	28 (6.1)	51 (3.6)
Drugs for peptic ulcer and gastro‐esophageal reflux disease (A02B), *n* yes (%)	26 (4.2)	26 (7.4)	17 (3.7)	69 (4.8)
Emollients and protectives (D02A), *n* yes (%)	1 (0.2)	1 (0.3)	5 (1.1)	7 (0.5)
High‐ceiling diuretics (C03C), *n* yes (%)	21 (3.4)	23 (6.5)	76 (16.7)	120 (8.4)
Hypnotics and sedatives (N05C), *n* yes (%)	37 (6.0)	22 (6.3)	91 (20.0)	150 (10.5)
Lipid modifying agent/plain (C10A), *n* yes (%)	14 (2.3)	1 (0.3)	0 (0)	15 (1.1)
Opioids (N02A), *n* yes (%)	20 (3.2)	12 (3.4)	29 (6.4)	61 (4.3)
Other analgesics and antipyretics (N02B), *n* yes (%)	99 (16.0)	115 (32.7)	174 (38.2)	388 (27.2)
Selective calcium channel blockers with mainly vascular effects–dihydropyridines (C08C), *n* yes (%)	10 (1.6)	1 (0.3)	0 (0)	11 (0.8)
Thyroid preparations (H03A), *n* yes (%)	28 (4.5)	15 (4.3)	23 (5.0)	66 (4.6)
Urologicals (G04B), *n* yes (%)	2 (0.3)	10 (2.8)	3 (0.7)	15 (1.1)
Vitamin B12 and folic acid (B03B), *n* yes (%)	8 (1.3)	19 (5.4)	33 (7.2)	60 (4.2)

*Note*: *M* (SD) = mean and standard deviation. *n* (%) = number of observations and percentage. The level of FI was multiplied by 100 to facilitate interpretation, and the estimates represent increments of 1 in these measures.

### 3.1 Medication effects

**FIGURE 1:**
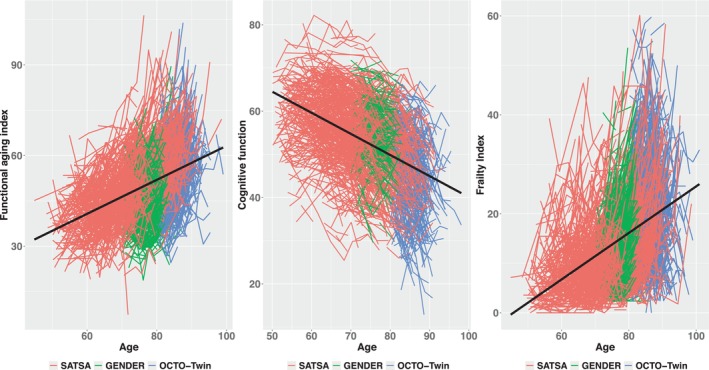
Functional aging index, cognitive function, and frailty index values over age (during all study period, separated by cohort). The functional aging index (FAI, *n* = 1191) has four domains: sensory functioning (hearing and vision), lung function, grip strength (corrected for sex), and gait. A higher score means worse performance/less ability. The cognitive function (COG, *n* = 1094) consists of a general cognitive ability measure (score) composed of distinct types of cognitive tests covering processing speed, verbal and spatial abilities, and memory (episodic and working memory). Higher values, better cognitive function. The frailty index (FI, *n* = 1361) was based on the accumulation of deficits approach. The index ranges from 0 to 1.0, with values closer to one representing more frailty. The level of FI was multiplied by 100 to facilitate interpretation. Each line in all graphs represents the participant's measurements over age in different IPTs. The black line represents a smoothing trend‐line considering linear model as a method. SATSA participants' age varies between 45 and 99 years. For GENDER, it varies between 69 and 88 years. Lastly, for OCTO‐Twin, it varies between 79 and 100 years. For FAI and FI, the graphs show increasing values of the indexes over age. The COG graph shows decreasing values of cognitive function over age.

During the study period, for all three cohorts (SATSA, GENDER, and OCTO‐Twin), the FAI and FI increased with age, while COG declined with age (Figure 1, Figure S4–S6). The mean years of follow‐up for FAI and FI samples were 8.7 years, and for COG it was 9.5 years (Table S11).

In the cGEE models for the FAI, 13 drugs reached *p* ≤ 0.15 to be included in Model 2. Anti‐inflammatory and antirheumatic products/nonsteroids (M01A), drugs for constipation (A06A), high‐ceiling diuretics (C03C), opioids (N02A), other analgesics and antipyretics (N02B), and vitamin B12 and folic acid (B03B) were not statistically significant after adjustments for age, CCI, smoking, BMI, number of drugs, and adjustments from the other drug classes (C09A, N06A, B01A, C07A, A12A, N05C, and C08C). Ace inhibitors/plain (*β* = 1.26, se = 0.54; effect size = 0.126), antidepressants (*β* = 2.38, se = 0.60; effect size = 0.238), antithrombotic agents (*β* = 1.00, se = 0.41; effect size = 0.1), beta‐blocking agents (*β* = 1.22, se = 0.42; effect size = 0.122), calcium (*β* = 1.45, se = 0.54; effect size = 0.145), as well as hypnotics and sedatives (*β* = 1.14, se = 0.52; effect size = 0.114) were associated with higher values of FAI after adjustments (worsening). Selective calcium channel blockers with mainly vascular effects—dihydropyridines (*β* = −1.29, se = 0.52; effect size = −0.129) were associated with lower values in the measurement after adjustments (improvement) (Table 2). In Bonferroni corrected results, only antidepressants and beta‐blocking agents remained significant (Table S6).

**TABLE 2:** Conditional generalized estimating equation models (cGEE) for drug effect on functional biomarkers of aging. SATSA, GENDER, and OCTO‐Twin.

	Functional aging index (*n* = 1191)		Cognitive function (*n* = 1094)		Frailty index (*n* = 1361)	
	Model 1	Model 2	Model 1	Model 2	Model 1	Model 2
Drugs (ATC code)	Estimates (95% CI)	Estimates (95% CI)	Estimates (95% CI)	Estimates (95% CI)	Estimates (95% CI)	Estimates (95% CI)
Ace inhibitors/plain (C09A)	2.03 (1.02, 3.05)*	**1.26 (0.20, 2.32)**	−0.26 (−0.84, 0.33)		2.02 (1.34, 2.69)*	**1.15 (0.50, 1.80)**
Adrenergics/inhalants (R03A)	0.81 (−1.34, 2.96)		0.82 (0.12, 1.51) *	**0.89 (0.16, 1.62)**	1.21 (−0.32, 2.74) *	0.53 (−0.76, 1.82)
Angiotensin ii receptor blockers (arbs)/plain (C09C)	−0.18 (−1.43, 1.06)		0.31 (−0.31, 0.92)		−0.20 (−1.03, 0.62)	
Antidepressants (N06A)	2.90 (1.70, 4.10) *	**2.38 (1.21, 3.55)**	−1.06 (−1.77, −0.36) *	**−0.90 (−1.67, −0.14)**	2.37 (1.14, 3.60) *	**1.59 (0.39, 2.79)**
Anti‐inflammatory and antirheumatic products/nonsteroids (M01A)	−0.60 (−1.29, 0.10) *	−0.54 (−1.25, 0.18)	−0.00 (−0.41, 0.40)		0.04 (−0.59, 0.68)	
Antithrombotic agents (B01A)	1.83 (1.10, 2.56) *	**1.00 (0.19, 1.81)**	−0.82 (−1.26, −0.39) *	**−0.84 (−1.33, −0.35)**	1.78 (1.23, 2.33) *	**0.75 (0.16, 1.34)**
Beta‐blocking agents (C07A)	1.73 (0.90, 2.56) *	**1.22 (0.39, 2.06)**	0.53 (0.08, 0.98) *	**0.69 (0.20, 1.18)**	1.87 (1.27, 2.46) *	**1.12 (0.55, 1.70)**
Blood glucose lowering drugs/excl. Insulins (A10B)	0.65 (−0.77, 2.07)		−0.91 (−1.87, 0.05) *	−0.73 (−1.68, 0.22)	0.20 (−0.79, 1.19)	
Calcium (A12A)	1.83 (0.71, 2.94) *	**1.45 (0.39, 2.51)**	0.22 (−0.35, 0.78)		1.72 (0.75,2.69) *	**0.99 (0.09, 1.88)**
Drugs for constipation (A06A)	1.55 (−0.08, 3.19) *	0.64 (−1.00, 2.28)	−0.58 (−1.41, 0.25)		1.01 (−0.05, 2.07) *	−0.12 (−1.11, 0.87)
Drugs for peptic ulcer and gastro‐esophageal reflux disease (A02B)	−0.05 (−0.97, 0.88)		0.20 (−0.29, 0.70)		1.25 (0.46, 2.05) *	0.41 (−0.34, 1.16)
Emollients and protectives (D02A)	0.40 (−1.49, 2.28)		0.13 (−0.84, 1.10)		0.59 (−1.40, 2.58)	
High‐ceiling diuretics (C03C)	1.95 (1.04, 2.86) *	0.93 (−0.04, 1.90)	−0.32 (−0.84, 0.20)		1.86 (1.05,2.67) *	0.50 (−0.29, 1.28)
Hypnotics and sedatives (N05C)	1.95 (0.92, 2.98) *	**1.14 (0.12, 2.16)**	−0.11 (−0.77, 0.55)		2.01 (1.22, 2.79) *	**1.10 (0.28, 1.91)**
Lipid modifying agent/plain (C10A)	0.16 (−0.89, 1.21)		0.55 (0.05, 1.06) *	**0.82 (0.31, 1.33)**	−0.09 (−0.76, 0.58)	
Opioids (N02A)	1.53 (0.49, 2.56) *	0.95 (−0.08, 1.99)	0.22 (−0.30, 0.74)		0.35 (−0.42, 1.13)	
Other analgesics and antipyretics (N02B)	0.71 (0.07, 1.34) *	0.52 (−0.17, 1.22)	−0.32 (−0.69, 0.04) *	−0.30 (−0.67, 0.07)	0.83 (0.40, 1.26) *	0.45 (−0.02, 0.91)
Selective calcium channel blockers with mainly vascular effects–dihydropyridines (C08C)	−0.95 (−1.95, 0.04) *	**−1.29 (−2.31, −0.28)**	0.31 (−0.19, 0.81)		0.16 (−0.54, 0.86)	
Thyroid preparations (H03A)	−0.27 (−2.19, 1.65)		0.06 (−0.98, 1.10)		0.48 (−0.79, 1.76)	
Urologicals (G04B)	−1.27 (−3.20, 0.65)		−0.58 (−1.74, 0.58)		1.35 (−0.28, 2.98) *	0.79 (−0.70, 2.28)
Vitamin B12 and folic acid (B03B)	0.76 (−0.22, 1.73) *	0.22 (−0.83, 1.26)	−0.04 (−0.70, 0.62)		0.47 (−0.28, 1.23)	

*Note*: The functional aging index (FAI) has four domains: sensory functioning (hearing and vision), lung function, grip strength (corrected for sex), and gait. A higher score means worse performance/less ability. The cognitive function (COG) consists of a general cognitive ability measure (score) composed of distinct types of cognitive tests covering processing speed, verbal and spatial abilities, and memory (episodic and working memory). The measure was created from a PCA, with T‐scoring (*M* = 50, SD = 10) on the created component scores and mean‐adjusted by sex. Higher values, better cognitive function. The frailty index (FI) was based on the accumulation of deficits approach. The index ranges from 0 to 1.0, with values more close to one representing more frail. The level of FI was multiplied by 100 to facilitate interpretation, and the estimates represent increments of 1 in these measures. Model 1: adjusted for age. Model 2: included all drugs with *p* ≤ 0.15 in model 1, adjusted for age, Charlson Comorbidity Index, smoking, body mass index, and number of drugs taken. All models were a bootstrap with resampling of twin pairs for 10,000 times. Bold values are significant medications after adjustments.

Abbreviation: CI, confidence interval.

* Medications with *p* ≤ 0.15 in Model 1.

For COG, seven drugs reached *p* ≤ 0.15 to be included in Model 2. Blood glucose lowering drugs/excl. Insulins (A10B) and other analgesics and antipyretics (N02B) were not statistically significant after adjustments for age, CCI, smoking, BMI, number of drugs, and other drug classes (R03A, N06A, B01A, C07A, and C10A). Adrenergics/inhalants (*β* = 0.89, se = 0.37; effect size = 0.089), beta‐blocking agents (*β* = 0.69, se = 0.25; effect size = 0.069), and lipid‐modifying agent/plain (*β* = 0.82, se = 0.26; effect size = 0.082) were associated with higher values in the cognitive ability measure after adjustments (improvement), while antidepressants (*β* = −0.90, se = 0.39; effect size = −0.090) and antithrombotic agents (*β* = −0.84, se = 0.25; effect size = −0.084) were associated with lower values in the cognitive ability measure after adjustments (worsening) (Table 2). In Bonferroni corrected results, only antithrombotic agents, beta‐blocking agents, and lipid‐modifying agent/plain remained significant (Table S6).

For the FI, 12 drugs reached *p* ≤ 0.15 to be included in Model 2. Adrenergics/inhalants (R03A), drugs for constipation (A06A), drugs for peptic ulcer and gastro‐esophageal reflux disease (A02B), high‐ceiling diuretics (C03C), other analgesics and antipyretics (N02B), and urologicals (G04B) were not statistically significant after full adjustments (age, CCI, smoking, BMI, number of drugs, C09A, N06A, B01A, C07A, A12A, and N05C). Ace inhibitors/plain (*β* = 1.15, se = 0.33), antidepressants (*β* = 1.59, se = 0.61), antithrombotic agents (*β* = 0.75, se = 0.30), beta‐blocking agents (*β* = 1.12, se = 0.29), calcium (*β* = 0.99, se = 0.46), and hypnotics and sedatives (*β* = 1.10, se = 0.41) were associated with higher values in the FI after adjustments (worsening). No FI decrements were found (Table 2). In Bonferroni corrected results, only ace inhibitors/plain and beta‐blocking agents remained significant (Table S6).

Separated analyses by sex were conducted. The results are presented in Tables S6–S8. Overall, Angiotensin ii receptor blockers (arbs)/plain (*β* = −1.92, se = 0.77; effect size = −0.192), anti‐inflammatory and antirheumatic products/non‐steroids (*β* = −1.74, se = 0.62; effect size = −0.174), blood glucose lowering drugs/excl. Insulins (*β* = −2.12, se = 0.96; effect size = −0.212), and selective calcium channel blockers with mainly vascular effects—dihydropyridines (*β* = −1.79, se = 0.77; effect size = −0.179) were associated with lower values in men's FAI (improvement). Emollients and protectives (*β* = 1.74, se = 0.55; effect size = 0.174) and lipid‐modifying agents/plain (*β* = 1.21, se = 0.34; effect size = 0.121) contributed to higher values in men's COG (improvement). Adrenergics/inhalants (*β* = 0.86, se = 0.42; effect size = 0.086), beta‐blocking agents (*β* = 1.14, se = 0.35; effect size = 0.114) and selective calcium channel blockers with mainly vascular effects—dihydropyridines (*β* = 0.90, se = 0.35; effect size = 0.090) showed higher values in women's COG (improvement). Angiotensin ii receptor blockers (arbs)/plain (*β* = −0.11, se = 0.05) contributed to lower values in men's FI (improvement).

## 4 DISCUSSION

In this study, we investigated the effects of the most common medications used in the Swedish older population on functional biomarkers. We found that selective calcium channel blockers with mainly vascular effects (dihydropyridines) were associated with lower values in FAI composed of measurements of hearing, vision, lung function, grip strength, and gait. In addition, adrenergics/inhalants and lipid‐modifying agents/plain were associated with higher values in COG. Despite beta‐blocking agents having apparently positive results on cognition, they were also associated with increased difficulties in FAI and FI over time.

We found that selective calcium channel blockers with mainly vascular effects (dihydropyridines) were associated with an improvement in the FAI. A limited number of scientific reports have examined the effect of these medications on functional status, and their results are mixed (Agostini et al., 2007; Baptista et al., 2018; Fragoso et al., 2020; Simon et al., 2015).

Similarly to our findings, Baptista et al. conducted an intervention study over 2 years to evaluate the effects of physical exercise and antihypertensive medications on functional status (*n* = 96 individuals). The medications were diuretics, calcium channel blockers (CCB), and beta blockers. They found that CCB were related to decreasing total cholesterol and improvements in physical functioning/physical limitations. However, they performed a non‐randomized study with no control group. Also, one of the interventions was physical activity (an exercise training program). As such, the effects could relate to physical activity or medication, or both (Baptista et al., 2018). Simon et al. (2015) explained in their review that angiotensin‐converting enzyme inhibitors and angiotensin receptor blockers might benefit muscle strength and endothelial function. However, to date, we found no consistent results of the effects of CCB on physical functioning.

The effects of the use of statins on cognitive function are also ambiguous in the literature (Alsubaie et al., 2022). However, our findings corroborate the results of observational studies presented by a systematic review (Adhikari et al., 2021), in which the use of statins was associated with a lower risk of cognitive impairment. In Randomized Controlled Trials, there was no difference in cognitive decline when participants were treated with rosuvastatin, pravastatin, and simvastatin compared to placebo groups. However, these trials had a short follow‐up time (Adhikari et al., 2021). Another systematic review showed that statins contribute to reducing dementia risk. Nevertheless, consistent benefits were observed only for simvastatin and atorvastatin (Zhu et al., 2018).

Lipids (phospholipids, sphingolipids, glycerolipids, fatty acids, sterols, and cholesterol) are essential for brain functionality and composition. In diseases related to cognitive decline, such as Alzheimer's disease, alterations in lipid metabolism (e.g., dysregulated sphingolipid metabolism and elevated levels of free fatty acids and cholesterol) are present and related to several dysfunctions, for example, neurotoxicity, higher levels of pro‐inflammatory fatty acids (e.g., arachidonic acid), oxidative stress, and mitochondrial dysfunction (Yin, 2023). The benefits of statins on cognition may be due to pleiotropic effects, such as anti‐inflammatory and antioxidant properties. The use of statins could contribute to lipid metabolism regulation and to reducing Aβ amyloid formation, providing neuroprotection (Alsubaie et al., 2022; Lamon‐Fava, 2013; Yin, 2023).

The effects of adrenergics/inhalants on cognitive function are unclear. A few animal studies suggest a relationship between β‐2‐adrenoreceptor agonists and improvements in cognitive function. The possible mechanisms of action are suggested to occur through lactate metabolism (improving learning and memory) (Dong et al., 2017) and improvement of mitochondrial dysfunction (Chai et al., 2022). However, their effects and long‐term use were not fully explored.

The functional biomarkers relate to other hallmarks of aging at the cellular level (López‐Otín et al., 2023). López‐Otín et al. (2023) suggested that cognitive function and other functional domains might be related to telomere attrition, epigenetic alterations, loss of proteostasis, disabled macroautophagy, deregulated nutrient‐sensing, chronic inflammation, and dysbiosis in animal models. Hence, the aforementioned positive pleiotropic effects of adrenergics/inhalants, lipid‐modifying agents/plain, and CCB on functional biomarkers could relate to better cellular and physiological performance.

Moreover, our results contribute to previous discussions on aging as a potential target for drug discovery in addition to disease‐based drug development (Couteur et al., 2022). Adrenergics/inhalants, lipid‐modifying agents/plain, and CCB may have an effect outside of their original indication, preserving an individual's functional independence through improvements in FAI and COG. However, it is yet to be discovered their effects on individuals without the diseases the drugs are prescribed for, proper dosage, and duration of treatment. Investments in aging as a pharmacological intervention target could prevent adverse aging outcomes, e.g., neurodegenerative diseases, and reduce future high healthcare costs.

For frailty, no medications had a beneficial effect in our main results; however, angiotensin ii receptor blockers (arbs)/plain might benefit men's FI (secondary analysis). The relationship between the effects of drug therapy and frailty is not established in the literature (Gutiérrez‐Valencia et al., 2018; Palmer et al., 2019), and not much interpretability can be extracted from our results since bias of indication might be present when a drug contributes to a biomarker of aging decline, and reverse causality is a possibility between FI items and the medications. The bias of indication occurs when the medication's effect appears to be associated with worsening frailty, but it is instead caused by the disease the drug was prescribed for (Joseph et al., 2014). In our study, reverse causality could occur when the diseases considered as items in the FI are associated with the medication prescription. Therefore, our FI results need to be further explored.

Knowledge about sex‐specific effects is crucial to understand the biological aging process. Men and women differ in many biological aging traits, such as telomere lengths, epigenetic age, and immunosenescence (Hägg & Jylhävä, 2021). Regarding functional biomarkers of aging, overall, men perform better in physical tests (Hägg & Jylhävä, 2021). Many studies exploring the effects of medications on physical functioning do not consider possible sex differences (Baptista et al., 2018; Fragoso et al., 2020; Zhang, Li, et al., 2020). In our research, women had more medications related to the decline of functional biomarkers. Meanwhile, men had more medications with a possible beneficial effect, and only one medication was beneficial for both sexes (selective calcium channel blockers with mainly vascular effects—dihydropyridines). These sex differences may be attributed to women having different pharmacokinetics (e.g., lower pH gastric fluid and lower basal metabolic rates), pharmacodynamics (e.g., lower renal clearance), adverse drug reactions compared to men, and different prescribing patterns (Skoog et al., 2014;Soldin & Mattison, 2009; Zucker & Prendergast, 2020). Ideally, sex differences should be considered when prescribing medications (e.g., weight‐adjusted dosing) (Soldin & Mattison, 2009; Zucker & Prendergast, 2020).

The strengths of this research are worth noting. We used harmonized information from three different longitudinal cohorts, which improves statistical power, and provides a relevant follow‐up period. Also, the harmonized information gives an “oversampling” of older adults aged 80 years or more, which may be underrepresented in previous studies. Another strength is that our exposures (medications) were measured at the same time‐point as the outcomes (FAI, COG, and FI) over time, meaning that, in our study the immortal time bias was less likely to occur (Yadav & Lewis, 2021). Finally, the cGEE model is adequate for longitudinal data with repeated measures from the same participant. When conditioning the cGEE on the individuals, the associations between medication use and functional biomarkers are implicitly adjusted for all time‐stable confounders (e.g., genes). This model's characteristic, in addition to adjustments for individual‐varying factors (e.g., chronological age, BMI, smoking status, number of medications used, and presence of diseases), provides more robust estimates and an opportunity for stronger causal claims.

Despite these strengths, the development of diseases is related to aging and increasing medication use (Zhang, Sundquist, et al., 2020). It should be noted that our results showing an effect of medications deteriorating functional aging, cognitive function, or frailty could be biased by confounding by indication, that is, the medications were prescribed for ill health and therefore associated with the outcomes. Conversely, the medications contributing to improvements in the functional biomarkers would be less biased by confounding by indication and could be closer to a possible real effect in decreasing biological aging. However, the generalizability of our study to the general population must be considered with caution since bias of indication cannot be completely eliminated, and some drug classes had limited availability on the Swedish market during the early waves of the studies, which may have contributed to the lower number of users as compared to other countries.

Yet another limitation is channeling biases (prescribing drug treatment, with similar indications, to groups of patients with different prognoses). In an attempt to consider both biases (indication and channeling), we adjusted our analysis for comorbidities and the number of drugs taken on each follow‐up. The estimated effects of adrenergics/inhalants and lipid‐modifying agents/plain on COG and the effects of channel blockers with mainly vascular effects—dihydropyridines on FAI were better estimated when adjusting for these two variables. Another limitation is the unavailability of the medication's dosage. Nevertheless, our research aimed to investigate which commonly used medications taken by older Swedish population could be beneficial for functional aging, cognitive function, and frailty. Future research exploring more detailed ATC level and dosages is recommended.

In addition to limitations, a complete case analysis assumes that the data are missing completely at random (MCAR), and multiple imputation and related strategies are an option under the slightly weaker assumption that data are missing at random (MAR). If the data are neither MCAR nor MAR, both complete case analysis and multiple imputation may give bias. In our analyses, we considered only complete data, and the exclusion of incomplete data can lead to a loss of precision and power (Sterne et al., 2009). Lastly, despite our caution in interpreting only the estimates contributing to improvements in the functional biomarkers and the direction of association of these estimates remained the same from the univariable to the multivariable models, the cGEE cannot deal with unobserved time‐varying confounders, and there is a possibility of Table 2 fallacy. Therefore, we suggest additional studies combining statistical and design‐based methods with stronger causal inference properties to confirm our findings and probable causal effects (Hammerton & Munafò, 2021).

To conclude, we identified possible beneficial effects of adrenergics/inhalants, lipid‐modifying agents/plain and calcium channel blockers—dihydropyridines on FAI and COG. This study discussed the possible rationale for why these medications could benefit biological aging. Future research evaluating the clinical value of these medications to prevent the decline of functional biomarkers, considering biological sex differences, is recommended.

## AUTHOR CONTRIBUTIONS

T.L.O. contributed to the conceptualization of the research, data curation, formal analysis, investigation, software, visualization, writing–original draft, and writing–review & editing. B.T. contributed supporting data curation, supporting formal analysis, and writing–review & editing. G.B. contributed supporting data curation and writing–review & editing. A.S. contributed supporting formal analysis and writing–review & editing. J.J. contributed with writing–review & editing. D.F. contributed supporting data curation, supporting methodology, and writing–review & editing. N.L.S. contributed supporting methodology and writing–review & editing. L.B.H. contributed with writing–review & editing. C.A.R. contributed supporting data curation, supporting methodology, and writing–review & editing. I.K. contributed with writing–review & editing. S.H. contributed to the conceptualization of the research, funding acquisition, methodology, project administration, resources, supervision, and writing–review & editing.

## FUNDING INFORMATION

This work was supported by the Swedish Research Council (grant numbers 2015‐03255, 2017‐00639, 2019‐01272, 2020‐06101, 2021‐00178, 2022‐01608); the National Institute on Aging (1R01AG067996); and Karolinska Institutet Foundations and Strategic Research Program in Epidemiology.

The funders of this research did not contribute to study design development, analysis, interpretation of data, and in the writing of the manuscript.

## CONFLICT OF INTEREST STATEMENT

The authors have no conflict of interest to declare.

## CONSENT TO PARTICIPATE

Informed consent was obtained from all individual participants included in the study.

## Supporting information


Data S1:

## Data Availability

The data cannot be shared publicly by the authors. The Swedish Adoption/Twin Study of Aging (SATSA) data are available from the National Archive of Computerized Data on Aging (NACDA) (2015) (https://www.icpsr.umich.edu/web/NACDA/studies/3843/summary). The Longitudinal Study of Gender Differences in Health Behavior and Health among Elderly (GENDER), and the Origins of Variance in the Oldest‐Old: Octogenarian Twins (OCTO‐Twin) data are available from The National E‐infrastructure for Aging Research (NEAR) (2023) (https://neardb.near‐aging.se/).
