# Supplementary material for: Effects from medications on functional biomarkers of aging in three longitudinal studies of aging in Sweden
Source: Aging Cell. 2024 Mar 1;23(6):e14132. doi: 10.1111/acel.14132 (PMC11296127; doi:10.1111/acel.14132)
Supplement: Supplementary file 1 — Data S1: [file ACEL-23-e14132-s001.docx]

**Supplementary Fig. 1. Data collection periods of the cohorts.**


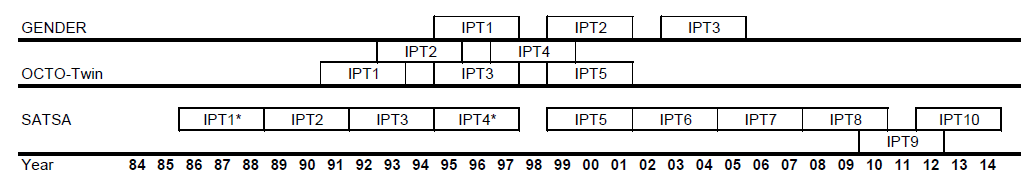


Note. IPT, in-person testing. *For the functional aging index and frailty index, there was no available measures in IPT1 and IPT4 for SATSA.

**Supplementary Table 1. Descriptive information on missing and complete data for functional aging index, cognitive function, and frailty index samples (during all study period. Total number of measurements: 4,615 for FAI, 4,383 for COG, and 5,396 for FI).**

|  | **Functional aging index** | | **Cognitive function** | | **Frailty index** | | |
| --- | --- | --- | --- | --- | --- | --- | --- |
| **Variable** | **Missing data (n=427)** | **Complete data (n=1,191)** | **Missing data (n= 557)** | **Complete data**  **(n = 1,094)** | **Missing data (n=286)** | **Complete data (n=1,361)** |  |
| Sex, n (%) |  |  |  |  |  |  |  |
| Men | 458 (31.5) | 1915 (41.5) | 670 (33.7) | 1759 (40.1) | 150 (36.1) | 2636 (48.9) |  |
| Women | 996 (68.5) | 2700 (58.5) | 1317 (66.3) | 2624 (59.9) | 266 (63.9) | 2760 (51.1) |  |
| Functional aging index, M (SD) | 54.9 (12.8) | 50.7 (12.0) | - | - | - | - |  |
| Cognitive function, M (SD) | - | - | 47.2 (11.2) | 51.7 (10.2) | - | - |  |
| Frailty index, M (SD) | - | - | - | - | 23 (12) | 15 (10) |  |
| Age (years), M (SD) | 82.3 (8.7) | 76.9 (9.0) | 82.6 (8.2) | 75.1 (9.5) | 84.1 (6.4) | 77.9 (9.2) |  |
| Charlson comorbidity index, M (SD) | 0.84 (1.29) | 0.49 (1.10) | 0.75 (1.23) | 0.43 (1.04) | 0.88 (1.34) | 0.52 (1.12) |  |
| Body mass index (kg/m^2^), M (SD) | 24.6 (4.6) | 25.9 (5.3) | 24.4 (4.1) | 25.9 (4.5) | 25.3 (3.8) | 25.7 (5.2) |  |
| Number of drugs, M (SD) | 4.02 (3.30) | 3.20 (2.72) | 2.40 (3.22) | 2.97 (2.59) | 2.00 (3.01) | 3.40 (2.84) |  |
| Smoke, n (%) |  |  |  |  |  |  |  |
| Not currently smoking | 542 (89.3) | 4142 (89.8) | 764 (86.5) | 3917 (89.4) | 245 (86.3) | 4847 (89.8) |  |
| Currently smoking | 65 (10.7) | 473 (10.2) | 119 (13.5) | 466 (10.6) | 39 (13.7) | 549 (10.2) |  |
| Ace inhibitors, plain (C09A), n (%) |  |  |  |  |  |  |  |
| Yes | 11 (1.3) | 304 (6.6) | 13 (0.7) | 267 (6.1) | 4 (0.5) | 342 (6.3) |  |
| Adrenergics, inhalants (R03A), n (%) |  |  |  |  |  |  |  |
| Yes | 27 (3.2) | 166 (3.6) | 32 (1.6) | 160 (3.7) | 10 (1.2) | 203 (3.8) |  |
| Angiotensin ii receptor blockers (arbs), plain (C09C), n (%) |  |  |  |  |  |  |  |
| Yes | 6 (0.7) | 142 (3.1) | 7 (0.4) | 143 (3.3) | 5 (0.6) | 152 (2.8) |  |
| Antidepressants (N06A), n (%) |  |  |  |  |  |  |  |
| Yes | 57 (6.7) | 219 (4.7) | 107 (5.4) | 147 (3.4) | 32 (3.9) | 288 (5.3) |  |
| Anti-inflammatory and antirheumatic products, non-steroids (M01A), n (%) |  |  |  |  |  |  |  |
| Yes | 59 (6.9) | 407 (8.8) | 66 (3.3) | 390 (8.9) | 27 (3.2) | 470 (8.7) |  |
| Antithrombotic agents (B01A), n (%) |  |  |  |  |  |  |  |
| Yes | 101 (11.9) | 1008 (21.8) | 175 (8.8) | 832 (19.0) | 40 (4.8) | 1185 (22.0) |  |
| Beta-blocking agents (C07A), n (%) |  |  |  |  |  |  |  |
| Yes | 64 (7.5) | 1003 (21.7) | 102 (5.1) | 907 (20.7) | 26 (3.1) | 1130 (20.9) |  |
| Blood glucose lowering drugs, excl. Insulins (A10B), n (%) |  |  |  |  |  |  |  |
| Yes | 49 (5.8) | 208 (4.5) | 73 (3.7) | 176 (4.0) | 27 (3.2) | 254 (4.7) |  |
| Calcium (A12A), n (%) |  |  |  |  |  |  |  |
| Yes | 15 (1.8) | 260 (5.6) | 32 (1.6) | 210 (4.8) | 4 (0.5) | 298 (5.5) |  |
| Drugs for constipation (A06A), n (%) |  |  |  |  |  |  |  |
| Yes | 176 (20.7) | 191 (4.1) | 222 (11.2) | 166 (3.8) | 96 (11.6) | 304 (5.6) |  |
| Drugs for peptic ulcer and gastro-oesophageal reflux disease (A02B), n (%) |  |  |  |  |  |  |  |
| Yes | 51 (6.0) | 405 (8.8) | 78 (3.9) | 351 (8.0) | 25 (3.0) | 481 (8.9) |  |
| Emollients and protectives (D02A), n (%) |  |  |  |  |  |  |  |
| Yes | 9 (1.1) | 33 (0.7) | 12 (0.6) | 28 (0.6) | 4 (0.5) | 46 (0.9) |  |
| High-ceiling diuretics (C03C), n (%) |  |  |  |  |  |  |  |
| Yes | 241 (28.3) | 578 (12.5) | 307 (15.5) | 487 (11.1) | 112 (13.5) | 803 (14.9) |  |
| Hypnotics and sedatives (N05C), n (%) |  |  |  |  |  |  |  |
| Yes | 182 (21.4) | 599 (13.0) | 262 (13.2) | 505 (11.5) | 92 (11.1) | 778 (14.4) |  |
| Lipid modifying agent, plain (C10A), n (%) |  |  |  |  |  |  |  |
| Yes | 4 (0.5) | 392 (8.5) | 9 (0.5) | 368 (8.4) | 2 (0.2) | 415 (7.7) |  |
| Opioids (N02A), n (%) |  |  |  |  |  |  |  |
| Yes | 135 (15.9) | 312 (6.8) | 176 (8.9) | 276 (6.3) | 73 (8.8) | 418 (7.7) |  |
| Other analgesics and antipyretics (N02B), n (%) |  |  |  |  |  |  |  |
| Yes | 287 (33.7) | 1012 (21.9) | 423 (21.3) | 952 (21.7) | 126 (15.2) | 1320 (24.5) |  |
| Selective calcium channel blockers with mainly vascular effects (C08C), n (%) |  |  |  |  |  |  |  |
| Yes | 5 (0.6) | 329 (7.1) | 9 (0.5) | 306 (7.0) | 3 (0.4) | 364 (6.7) |  |
| Thyroid preparations (H03A), n (%) |  |  |  |  |  |  |  |
| Yes | 34 (4.0) | 313 (6.8) | 40 (2.0) | 293 (6.7) | 8 (1.0) | 381 (7.1) |  |
| Urologicas (G04B), n (%) |  |  |  |  |  |  |  |
| Yes | 12 (1.4) | 63 (1.4) | 13 (0.7) | 54 (1.2) | 4 (0.5) | 80 (1.5) |  |
| Vitamin B12 and folic acid (B03B), n (%) |  |  |  |  |  |  |  |
| Yes | 114 (13.4) | 436 (9.4) | 152 (7.7) | 363 (8.3) | 38 (4.6) | 571 (10.6) |  |

Note: FAI: functional aging index. COG: cognitive function. FI: frailty index.

**Supplementary Fig. 2. Flow-chart of participants included in the analyses (SATSA, GENDER, and OCTO-Twin).**

**
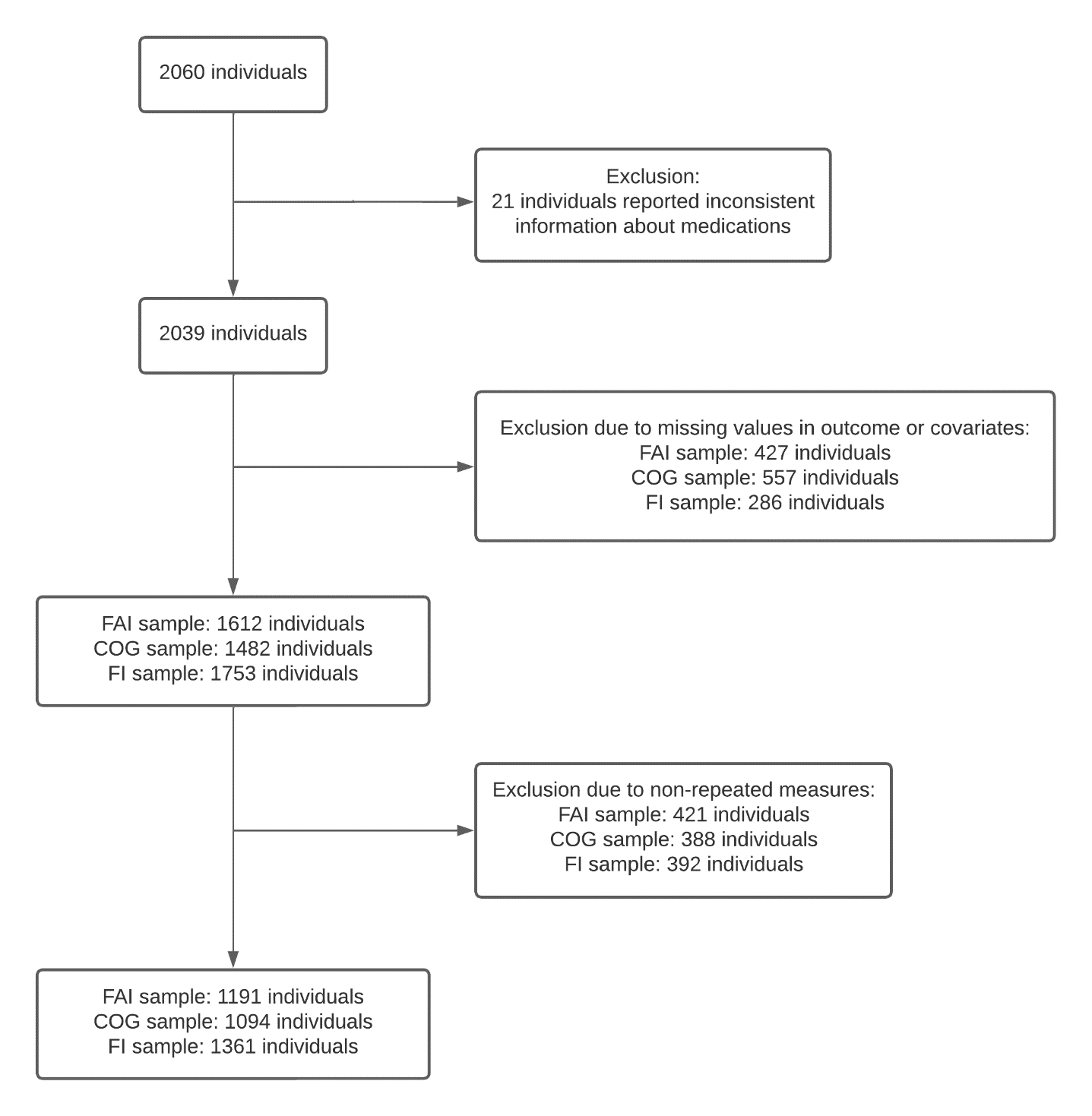
**

Note. The sample size is different according to the missing values in the outcomes or time-varying confounders. FAI: functional aging index. COG: cognitive function. FI: frailty index.

**Criteria for selecting the exposure variables**

To check the most common medications, we accessed the Statistics Sweden website (https://sdb.socialstyrelsen.se/if_lak/val.aspx) and selected annual information for Sweden, age 65 years or higher, gender (separately for men and women), all available third level of ATC codes, and the number of patients. The medications do not differ if selecting the number of patients (number of people who collected a medicine at least once during the year or month) or patients/1000 inhabitants (number of patients, divided by the total population of the relevant group (age group, county, etc.)). We excluded sex-specific (G03C: estrogens and G04C: drugs used in benign prostatic hypertrophy) and short-term medications (J01C: beta-lactam antibacterials, penicillins) from our analysis. Supplementary Table 2 and 3 show the number of people who collected medicine at least once during the year or month for men and women, respectively, for 2022.

**Supplementary Table 2. Twenty most common medications taken by individuals with age higher than 65 years in 2022, male sex.**

| **ATC code – Pharmacological subgroup name** | **Number of individuals** |
| --- | --- |
| B01A Antithrombotic agents | 458660 |
| C10A Lipid modifying agent/plain | 456046 |
| C07A Beta-blocking agents | 354567 |
| C08C Selective calcium channel blockers with mainly vascular effects | 304333 |
| N02B Other analgesics and antipyretics | 272071 |
| A02B Drugs for peptic ulcer and gastro-oesophageal reflux disease | 238820 |
| C09C Angiotensin ii receptor blockers (arbs)/plain | 237512 |
| C09A Ace inhibitors/plain | 199216 |
| A10B Blood glucose lowering drugs/excl. Insulins | 198049 |
| G04C Drugs used in benign prostatic hypertrophy | 186269 |
| B03B Vitamin B12 and folic acid | 170880 |
| A06A Drugs for constipation | 165200 |
| J01C Beta-lactam antibacterials, penicillins | 133588 |
| N06A Antidepressants | 131518 |
| N02A Opioids | 129423 |
| N05C Hypnotics and sedatives | 129312 |
| G04B Urologicas | 122625 |
| C03C High-ceiling diuretics | 110571 |
| D02A Emollients and protectives | 109739 |
| M01A Anti-inflammatory and antirheumatic products/non-steroids | 104136 |

**Supplementary Table 3. Twenty most common medications taken by individuals with age higher than 65 years in 2022, female sex.**

| **ATC code – Pharmacological subgroup name** | **Number of individuals** |
| --- | --- |
| N02B Other analgesics and antipyretics | 437334 |
| C10A Lipid modifying agent/plain | 395053 |
| B01A Antithrombotic agents | 381250 |
| C07A Beta-blocking agents | 380795 |
| A02B Drugs for peptic ulcer and gastro-oesophageal reflux disease | 318947 |
| C08C Selective calcium channel blockers with mainly vascular effects | 312990 |
| C09C Angiotensin ii receptor blockers (arbs)/plain | 276723 |
| N06A Antidepressants | 252776 |
| N05C Hypnotics and sedatives | 243121 |
| A06A Drugs for constipation | 238133 |
| B03B Vitamin B12 and folic acid | 211562 |
| A12A Calcium | 199424 |
| G03C Estrogens | 199024 |
| J01C Beta-lactam antibacterials, penicillins | 193803 |
| N02A Opioids | 186802 |
| H03A Thyroid preparations | 184024 |
| C09A Ace inhibitors/plain | 160701 |
| R03A Adrenergics/inhalants | 159759 |
| D02A Emollients and protectives | 147648 |
| M01A Anti-inflammatory and antirheumatic products/non-steroids | 143184 |

**Supplementary Fig. 3-A. Drug use variation during the study period (SATSA, GENDER, and OCTO-Twin).**

**
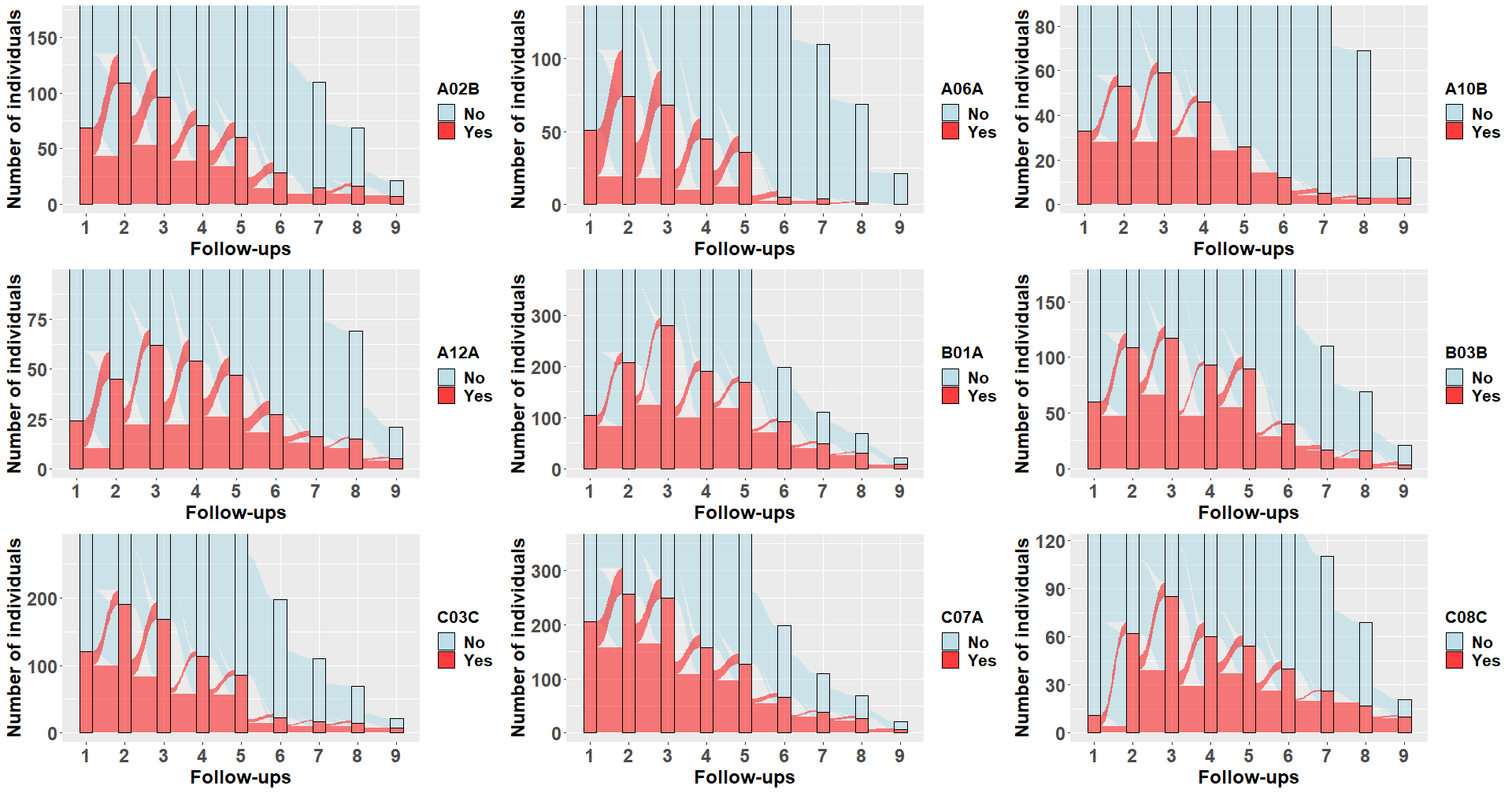
**

Note. The plots show only the participants with drug use variation during the study period. The y-axis has 1,427 individuals; however, not represented in the graphs. A02B: Drugs for peptic ulcer and gastro-oesophageal reflux disease. A06A: Drugs for constipation. A10B: Blood glucose lowering drugs/excl. Insulins. A12A: Calcium. B01A: Antithrombotic agents. B03B: Vitamin B12 and folic acid. C03C: High-ceiling diuretics. C07A: Beta-blocking agents. C08C: Selective calcium channel blockers with mainly vascular effects (dihydropyridines).

**Supplementary Fig. 3-B. Drug use variation during the study period (SATSA, GENDER, and OCTO-Twin).**

**
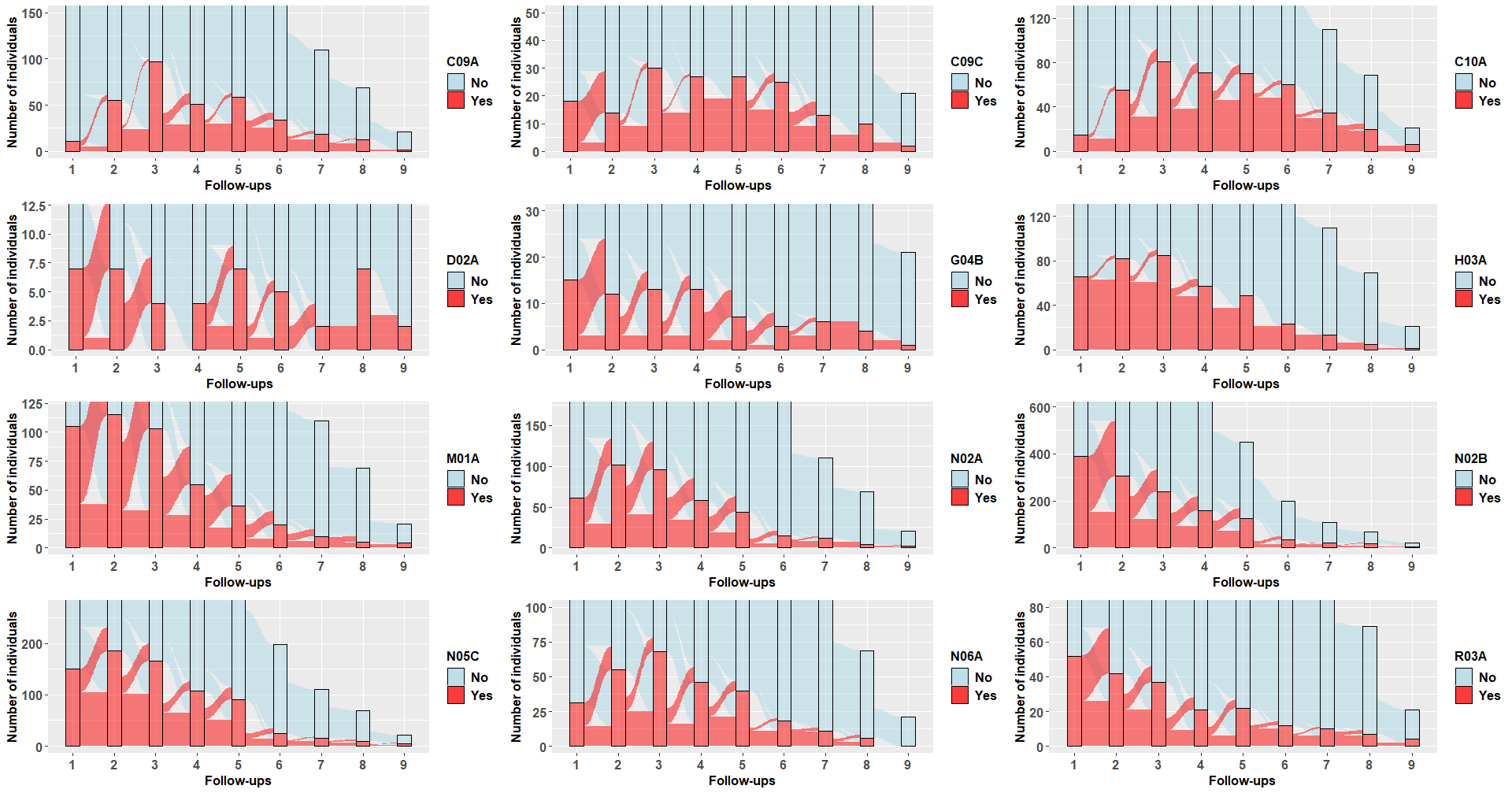
**

Note. The plots show only the participants with drug use variation during the study period. The y-axis has 1,427 individuals; however, not represented in the graphs. C09A: Ace inhibitors/plain. C09C: Angiotensin ii receptor blockers (arbs)/plain. C10A: Lipid modifying agent/plain. D02A: Emollients and protectives. G04B: Urologicas. H03A: Thyroid preparations. M01A: Anti-inflammatory and antirheumatic products/non-steroids. N02A: Opioids. N02B: Other analgesics and antipyretics. N05C: Hypnotics and sedatives. N06A: Antidepressants. R03A: Adrenergics/inhalants.

**Supplementary Table 4. Correlations among individual biomarkers that compose functional aging index and factor loadings at each wave (adapted from** Finkel et al., 2019**).**

|  | Wave1 | Wave2 | Wave3 | Wave4 | Wave5 | Wave6 | Wave7 | Wave8 |
| --- | --- | --- | --- | --- | --- | --- | --- | --- |
| Variables | SATSA  OCTO-Twin  GENDER | SATSA  OCTO-Twin  GENDER | SATSA  OCTO-Twin  GENDER | SATSA  OCTO-Twin | SATSA  OCTO-Twin | SATSA | SATSA | SATSA |
| N | 1695 | 1561 | 1436 | 948 | 763 | 377 | 448 | 265 |
| *Correlations* |  |  |  |  |  |  |  |  |
| Grip x gait | .33 | .32 | .33 | .28 | .31 | .33 | .42 | .44 |
| Grip x sense | .11 | .11 | .14 | .20 | .21 | .21 | .26 | .27 |
| Grip x PEF | .32 | .19 | .25 | .27 | .17 | .32 | .30 | .23 |
| Gait x sense | .15 | .18 | .22 | .25 | .17 | .18 | .24 | .16 |
| Gait x PEF | .34 | .31 | .28 | .30 | .11 | .34 | .22 | .22 |
| Sense x PEF | .16 | .14 | .15 | .15 | .14 | .15 | .20 | .15 |
| *Factor Loadings* | |  |  |  |  |  |  |  |
| Grip | .68 | .63 | .66 | .67 | .77 | .69 | .78 | .75 |
| Gait | .73 | .76 | .74 | .71 | .68 | .70 | .72 | .73 |
| Sense | .36 | .41 | .51 | .55 | .51 | .53 | .52 | .57 |
| PEF | .75 | .70 | .63 | .67 | .69 | .69 | .53 | .47 |
| Variance explained | 42% | 40% | 41% | 43% | 43% | 43% | 42% | 41% |

Note. Sense: self-reported hearing and self-reported vision. PEF: peak expiratory flow.

**Supplementary Table 5. Items used to calculate the Rockwood frailty index in SATSA, GENDER, and OCTO-Twin (adapted from Bai et al. 2021).**

| **Item** | **SATSA** | **OCTO-Twin** | **GENDER** |
| --- | --- | --- | --- |
| Allergies/allergic manifestations | X | X |  |
| Anemia | X | X |  |
| Arthritis | X | X |  |
| Asthma | X | X |  |
| Cancer or leukemia | X |  | X |
| Cataracts | X | X | X |
| Cerebral hemorrhage or blood clot in brain | X | X |  |
| Chest pain | X | X | X |
| Chronic bronchitis or emphysema | X | X |  |
| Circulation problems in arms or legs | X | X | X |
| Depressions | X |  | X |
| Diabetes | X | X | X |
| Dizziness | X | X |  |
| Dress and undress | X |  | X |
| Eczema |  | X | X |
| Epilepsy |  |  | X |
| Feel happy | X |  |  |
| Feel tired | X |  |  |
| Gall bladder |  | X | X |
| Gastric ulcer | X | X | X |
| Get in and out of bed | X |  | X |
| Glaucoma |  | X | X |
| Goiter or other gland problems | X | X | X |
| Gout |  |  | X |
| Grocery shopping | X |  | X |
| Handle small things with your fingers |  |  | X |
| Health limits activities | X | X | X |
| Hearing acuity | X | X |  |
| Heart attack |  | X | X |
| Heart failure | X | X | X |
| Herpes |  | X |  |
| Hip joint impairment |  | X | X |
| Housework | X |  | X |
| Hypertension | X | X | X |
| Insomnia |  | X | X |
| Keep body fit |  | X |  |
| Kidney disease | X | X | X |
| Liver disease |  |  | X |
| Loneliness | X | X | X |
| Manage medications | X |  | X |
| Manage money | X |  |  |
| Migraine |  | X | X |
| Neck pain |  | X |  |
| Osteoporosis | X | X | X |
| Persistent cough | X |  |  |
| Picking something up from the floor |  |  | X |
| Prepare meals | X |  |  |
| Psychological problems |  | X |  |
| Rheumatoid arthritis | X | X | X |
| Sciatica | X | X | X |
| Self-grooming | X |  |  |
| Self-reported general health | X | X | X |
| Shoulder pain |  | X |  |
| Shower and bathe | X | X | X |
| Speech impairment |  | X | X |
| Stroke |  |  | X |
| Travel further distances | X |  | X |
| Trouble getting to toilet in time | X | X | X |
| Use telephone | X |  | X |
| Vascular spasm in leg |  | X | X |
| Vision acuity | X | X |  |
| Walking | X | X | X |
| Total number of items | 42 | 41 | 42 |

**Supplementary Table 6. Conditional generalized estimating equation models (cGEE) for drug effect on functional biomarkers of aging, considering Bonferroni correction for model 2 estimates. SATSA, GENDER, and OCTO-Twin.**

|  | **Functional Aging Index (n=1,191)** | | **Cognitive function (n=1,094)** | | **Frailty Index (n=1,361)** | |
| --- | --- | --- | --- | --- | --- | --- |
| Drugs (ATC code) | **Estimates (model 2)** | ***p-value (<0.004)** | **Estimates**  **(model 2)** | ***p- value (<0.007)** | **Estimates**  **(model 2)** | ***p-value (<0.004)** |
| Ace inhibitors/plain (C09A) | 1.26 | 0.02 |  |  | **1.15** | **0.00049** |
| Adrenergics/inhalants (R03A) |  |  | 0.89 | 0.017 | 0.53 | 0.42 |
| Angiotensin ii receptor blockers (arbs)/plain (C09C) |  |  |  |  |  |  |
| Antidepressants (N06A) | **2.38** | **0.00007** | -0.90 | 0.02 | 1.59 | 0.0091 |
| Anti-inflammatory and antirheumatic products/non-steroids (M01A) | -0.54 | 0.14 |  |  |  |  |
| Antithrombotic agents (B01A) | 1.00 | 0.016 | **-0.84** | **0.00084** | 0.75 | 0.013 |
| Beta-blocking agents (C07A) | **1.22** | **0.004** | **0.69** | **0.006** | **1.12** | **0.00013** |
| Blood glucose lowering drugs/excl. Insulins (A10B) |  |  | -0.73 | 0.13 |  |  |
| Calcium (A12A) | 1.45 | 0.0073 |  |  | 0.99 | 0.031 |
| Drugs for constipation (A06A) | 0.64 | 0.44 |  |  | -0.12 | 0.81 |
| Drugs for peptic ulcer and gastro-oesophageal reflux disease (A02B) |  |  |  |  | 0.41 | 0.28 |
| Emollients and protectives (D02A) |  |  |  |  |  |  |
| High-ceiling diuretics (C03C) | 0.93 | 0.061 |  |  | 0.50 | 0.21 |
| Hypnotics and sedatives (N05C) | 1.14 | 0.028 |  |  | 1.10 | 0.0081 |
| Lipid modifying agent/plain (C10A) |  |  | **0.82** | **0.0017** |  |  |
| Opioids (N02A) | 0.95 | 0.072 |  |  |  |  |
| Other analgesics and antipyretics (N02B) | 0.52 | 0.14 | -0.30 | 0.11 | 0.45 | 0.058 |
| Selective calcium channel blockers with mainly vascular effects - dihydropyridines (C08C) | -1.29 | 0.013 |  |  |  |  |
| Thyroid preparations (H03A) |  |  |  |  |  |  |
| Urologicas (G04B) |  |  |  |  | 0.79 | 0.30 |
| Vitamin B12 and folic acid (B03B) | 0.22 | 0.69 |  |  |  |  |

Note. The functional aging index (FAI) has four domains: sensory functioning (hearing and vision), lung function, grip strength (corrected for sex), and gait. A higher score means worse performance/less ability. The cognitive function (COG) consists of a general cognitive ability measure (score) composed of distinct types of cognitive tests covering processing speed, verbal and spatial abilities, and memory (episodic and working memory). The measure was created from a PCA, with T-scoring (M=50, SD=10) on the created component scores and mean-adjusted by sex. Higher values, better cognitive function. The frailty index (FI) was based on the accumulation of deficits approach. The index ranges from 0 to 1.0, with values more close to one representing more frail. The level of FI was multiplied by 100 to facilitate interpretation, and the estimates represent increments of 1 in these measures. Model 2: included all drugs with p-value ≤0.15 in model 1 (Table 2), adjusted for age, Charlson comorbidity index, smoking, body mass index, and number of drugs taken. All models were a bootstrap with resampling of twin pairs for 10,000 times. Bold values are significant medications after adjustments. * The value in parentheses represents the significance levels considering Bonferroni correction for each outcome.

**Supplementary Table 7. Conditional generalized estimating equation models (cGEE) for functional aging index, separated by sex. SATSA, GENDER, and OCTO-Twin (n=1,191).**

|  | **Functional aging index** | | | |
| --- | --- | --- | --- | --- |
|  | **Men** | | **Women** | |
|  | **Model 1** | **Model 2** | **Model 1** | **Model 2** |
| Drugs (ATC code) | Estimates 95% CI | Estimates 95% CI | Estimates 95% CI | Estimates 95% CI |
| Ace inhibitors, plain (C09A) | 1.36 (0.11,2.61) * | 0.03 (-1.35, 1.41) | 2.94 (1.44, 4.45) * | **2.26 (0.76, 3.75)** |
| Adrenergics, inhalants (R03A) | 2.72 (-1.68, 7.13) |  | -0.26 (-2.01, 1.49) |  |
| Angiotensin ii receptor blockers (arbs), plain (C09C) | -1.30 (-2.88, 0.29) * | **-1.92 (-3.42, -0.41)** | 0.71 (-1.03, 2.44) |  |
| Antidepressants (N06A) | 3.30 (1.18,5.42) * | **2.17 (0.03, 4.31)** | 2.67 (1.32, 4.02) * | **2.37 (1.02, 3.71)** |
| Anti-inflammatory and antirheumatic products, non-steroids (M01A) | -1.43 (-2.56, -0.29) * | **-1.74 (-2.96, -0.51)** | -0.23 (-1.06, 0.61) |  |
| Antithrombotic agents (B01A) | 2.11 (0.95,3.28) * | 0.84 (-0.29, 1.98) | 1.61 (0.74, 2.47) * | **1.04 (0.03, 2.06)** |
| Beta-blocking agents (C07A) | 2.63 (1.46,3.80) * | **1.92 (0.76, 3.07)** | 1.01 (-0.09, 2.11) * | 0.55 (-0.51, 1.61) |
| Blood glucose lowering drugs, excl. Insulins (A10B) | -1.82 (-3.36, -0.28) * | **-2.12 (-4.00, -0.25)** | 2.56 (0.58, 4.55) * | **2.24 (0.43, 4.05)** |
| Calcium (A12A) | 2.27 (-4.08, 8.62) |  | 1.91 (0.96, 2.86) * | **1.86 (0.83, 2.89)** |
| Drugs for constipation (A06A) | 1.50 (-1.36, 4.36) |  | 1.59 (-0.33, 3.50) * | 0.81 (-1.07, 2.69) |
| Drugs for peptic ulcer and gastro-oesophageal reflux disease (A02B) | 0.04 (-1.32, 1.39) |  | -0.08 (-1.27, 1.11) |  |
| Emollients and protectives (D02A) | -1.38 (-4.15, 1.39) |  | 2.41 (0.34, 4.48) * | 2.12 (-0.11, 4.34) |
| High-ceiling diuretics (C03C) | 3.20 (1.82,4.59) * | 1.07 (-0.54, 2.68) | 1.32 (0.21, 2.42) * | 1.02 (-0.15, 2.19) |
| Hypnotics and sedatives (N05C) | 1.38 (-0.61, 3.38) |  | 2.23 (1.09, 3.37) * | **1.91 (0.82, 2.99)** |
| Lipid modifying agent, plain (C10A) | 0.11 (-1.34, 1.56) |  | 0.13 (-1.19, 1.44) |  |
| Opioids (N02A) | 2.73 (0.66,4.80) * | 1.65 (-0.37, 3.67) | 0.98 (-0.14, 2.10) * | 0.74 (-0.34, 1.81) |
| Other analgesics and antipyretics (N02B) | 0.92 (-0.22, 2.06) * | 0.44 (-0.82, 1.70) | 0.61 (-0.14, 1.37) * | 0.63 (-0.19, 1.46) |
| Selective calcium channel blockers with mainly vascular effects (C08C) | -1.45 (-2.88, -0.01) * | **-1.79 (-3.33, -0.25)** | -0.54 (-1.76, 0.68) |  |
| Thyroid preparations (H03A) | 1.80 (-0.77, 4.37) |  | -0.58 (-2.65, 1.48) |  |
| Urologicas (G04B) | -0.70 (-3.87, 2.48) |  | -1.73 (-3.92, 0.45) * | -2.03 (-4.17, 0.10) |
| Vitamin B12 and folic acid (B03B) | 1.66 (0.50,2.82) * | 0.78 (-0.49, 2.05) | 0.03 (-1.31, 1.38) |  |

Note. The functional aging index has four domains: sensory functioning (hearing and vision), lung function, grip strength (corrected for sex), and gait. A higher score means worse performance/less ability. Model 1: adjusted for age. Model 2: included all drugs with p-value ≤0.15 in model 1, adjusted for age, Charlson comorbidity index, smoking, body mass index, and number of drugs taken. All models were a bootstrap with resampling of twin pairs for 10,000 times. Bold values are significant medications after adjustments. CI: confidence interval. *medications with p-value p≤0.15 in model 1.

**Supplementary Table 8. Conditional generalized estimating equation models (cGEE) for cognitive function, separated by sex. SATSA, GENDER, and OCTO-Twin (n=1,094).**

|  | **Cognitive function** | | | |
| --- | --- | --- | --- | --- |
|  | **Men** | | **Women** | |
|  | **Model 1** | **Model 2** | **Model 1** | **Model 2** |
| Drugs (ATC code) | Estimates 95% CI | Estimates 95% CI | Estimates 95% CI | Estimates 95% CI |
| Ace inhibitors, plain (C09A) | 0.18 (-0.35, 0.71) |  | -0.94 (-2.09, 0.21) * | -0.74 (-1.82, 0.34) |
| Adrenergics, inhalants (R03A) | 0.72 (-0.71, 2.16) |  | 0.88 (0.15, 1.61) * | **0.86 (0.04, 1.68)** |
| Angiotensin ii receptor blockers (arbs), plain (C09C) | -0.39 (-1.16, 0.37) |  | 0.74 (-0.10, 1.57) * | 0.70 (-0.13, 1.53) |
| Antidepressants (N06A) | 0.08 (-0.78, 0.94) |  | -1.65 (-2.65, -0.65) * | **-1.58 (-2.70, -0.46)** |
| Anti-inflammatory and antirheumatic products, non-steroids (M01A) | 0.06 (-0.54, 0.65) |  | -0.05 (-0.55, 0.46) |  |
| Antithrombotic agents (B01A) | -0.86 (-1.44, -0.27) * | **-0.73 (-1.42, -0.04)** | -0.86 (-1.45, -0.27) * | **-0.69 (-1.34, -0.04)** |
| Beta-blocking agents (C07A) | 0.06 (-0.51, 0.63) |  | 0.85 (0.24, 1.45) * | **1.14 (0.45, 1.82)** |
| Blood glucose lowering drugs, excl. Insulins (A10B) | 0.03 (-1.19, 1.24) |  | -1.66 (-2.90, -0.41) * | **-1.65 (-2.90, -0.41)** |
| Calcium (A12A) | 0.70 (-2.35, 3.75) |  | 0.27 (-0.31, 0.85) |  |
| Drugs for constipation (A06A) | -0.64 (-1.96, 0.69) |  | -0.55 (-1.57, 0.47) |  |
| Drugs for peptic ulcer and gastro-oesophageal reflux disease (A02B) | -0.07 (-0.69, 0.54) |  | 0.40 (-0.28, 1.07) |  |
| Emollients and protectives (D02A) | 1.66 (0.69, 2.63) * | **1.74 (0.66, 2.81)** | -1.28 (-2.27, -0.29) * | **-1.43 (-2.55, -0.31)** |
| High-ceiling diuretics (C03C) | -0.60 (-1.39, 0.19) * | -0.21 (-1.06, 0.64) | -0.17 (-0.82, 0.48) |  |
| Hypnotics and sedatives (N05C) | 0.08 (-0.60, 0.76) |  | -0.15 (-0.98, 0.69) |  |
| Lipid modifying agent, plain (C10A) | 0.78 (0.20, 1.36) * | **1.21 (0.55, 1.88)** | 0.22 (-0.56, 1.00) |  |
| Opioids (N02A) | 0.33 (-0.51, 1.17) |  | 0.16 (-0.48, 0.81) |  |
| Other analgesics and antipyretics (N02B) | -0.38 (-1.00, 0.25) |  | -0.29 (-0.73, 0.14) |  |
| Selective calcium channel blockers with mainly vascular effects (C08C) | -0.16 (-0.76, 0.45) |  | 0.69 (-0.03, 1.40) * | **0.90 (0.20, 1.60)** |
| Thyroid preparations (H03A) | 0.34 (-2.04, 2.72) |  | 0.11 (-1.04, 1.26) |  |
| Urologicas (G04B) | -1.57 (-2.91, -0.23) * | **-1.47 (-2.86, -0.08)** | 0.11 (-1.45, 1.67) |  |
| Vitamin B12 and folic acid (B03B) | 0.30 (-0.41, 1.00) |  | -0.30 (-1.26, 0.66) |  |

Note. The cognitive function consists of a general cognitive ability measure (score) composed of distinct types of cognitive tests covering processing speed, verbal and spatial abilities, and memory (episodic and working memory). The measure was created from a PCA, with T-scoring (M=50, SD=10) on the created component scores and mean-adjusted by sex. Higher values, better cognitive function. Model 1: adjusted for age. Model 2: included all drugs with p-value ≤0.15 in model 1, adjusted for age, Charlson comorbidity index, smoking, body mass index, and number of drugs taken. All models were a bootstrap with resampling of twin pairs for 10,000 times. Bold values are significant medications after adjustments. CI: confidence interval. *medications with p-value p≤0.15 in model 1.

**Supplementary Table 9. Conditional generalized estimating equation models (cGEE) for frailty index, separated by sex. SATSA, GENDER, and OCTO-Twin (n=1,361).**

|  | **Frailty index** | | | |
| --- | --- | --- | --- | --- |
|  | **Men** | | **Women** | |
|  | **Model 1** | **Model 2** | **Model 1** | **Model 2** |
| Drugs (ATC code) | Estimates 95% CI | Estimates 95% CI | Estimates 95% CI | Estimates 95% CI |
| Ace inhibitors, plain (C09A) | 0.31 (0.21, 0.41) * | **0.16 (0.06, 0.27)** | 0.14 (0.08,0.21) * | **0.10 (0.02,0.17)** |
| Adrenergics, inhalants (R03A) | 0.01 (-0.17, 0.18) |  | 0.21 (-0.04, 0.46) * | 0.13 (-0.09, 0.34) |
| Angiotensin ii receptor blockers (arbs), plain (C09C) | -0.10 (-0.20, 0.00) * | **-0.11 (-0.20, -0.01)** | 0.06 (-0.05, 0.18) |  |
| Antidepressants (N06A) | 0.39 (0.24,0.54) * | **0.30 (0.16, 0.45)** | 0.11 (-0.07, 0.29) |  |
| Anti-inflammatory and antirheumatic products, non-steroids (M01A) | 0.02 (-0.06, 0.11) |  | -0.01 (-0.10, 0.08) |  |
| Antithrombotic agents (B01A) | 0.18 (0.11,0.25) * | 0.03 (-0.05, 0.11) | 0.20 (0.12,0.27) * | **0.13 (0.05,0.20)** |
| Beta-blocking agents (C07A) | 0.19 (0.12, 0.26) * | **0.11 (0.04, 0.18)** | 0.19 (0.10,0.28) * | **0.12 (0.04,0.21)** |
| Blood glucose lowering drugs, excl. Insulins (A10B) | -0.05 (-0.16, 0.05) |  | 0.10 (-0.05, 0.25) |  |
| Calcium (A12A) | 0.11 (0.02, 0.21) * | 0.02 (-0.06, 0.11) | 0.21 (-0.02, 0.43) * | 0.14 (-0.06, 0.34) |
| Drugs for constipation (A06A) | 0.10 (-0.07, 0.27) |  | 0.12 (0.00, 0.24) * | 0.01 (-0.10, 0.11) |
| Drugs for peptic ulcer and gastro-oesophageal reflux disease (A02B) | 0.20 (0.11,0.30) * | **0.09 (0.00, 0.17)** | 0.05 (-0.07, 0.16) |  |
| Emollients and protectives (D02A) | 0.25 (0.05, 0.46) * | 0.15 (-0.04, 0.35) | -0.18 (-0.41, 0.05) * | -0.16 (-0.40, 0.08) |
| High-ceiling diuretics (C03C) | 0.27 (0.17, 0.38) * | **0.13 (0.03, 0.23)** | 0.12 (0.01,0.23) * | -0.02 (-0.13, 0.09) |
| Hypnotics and sedatives (N05C) | 0.19 (0.10,0.29) * | 0.08 (-0.02, 0.17) | 0.20 (0.09, 0.32) * | **0.12 (0.00,0.25)** |
| Lipid modifying agent, plain (C10A) | 0.00 (-0.08, 0.08) |  | 0.01 (-0.08, 0.10) |  |
| Opioids (N02A) | 0.06 (-0.04, 0.17) |  | 0.02 (-0.08, 0.13) |  |
| Other analgesics and antipyretics (N02B) | 0.08 (0.02,0.14) * | 0.02 (-0.05, 0.09) | 0.08 (0.03,0.14) * | **0.06 (0.00,0.12)** |
| Selective calcium channel blockers with mainly vascular effects (C08C) | -0.02 (-0.11, 0.07) |  | 0.05 (-0.04, 0.15) |  |
| Thyroid preparations (H03A) | -0.04 (-0.19, 0.11) |  | 0.15 (-0.03, 0.34) * | 0.12 (-0.07, 0.30) |
| Urologicas (G04B) | 0.06 (-0.14, 0.27) |  | 0.25 (0.05,0.44) * | **0.20 (0.02,0.39)** |
| Vitamin B12 and folic acid (B03B) | 0.10 (0.00, 0.21) * | 0.02 (-0.07, 0.12) | 0.00 (-0.10, 0.09) |  |

Note. The frailty index (FI) was based on the accumulation of deficits approach. The index ranges from 0 to 1.0, with values more close to one representing more frail. The level of FI was multiplied by 100 to facilitate interpretation, and the estimates represent increments of 1 in these measures. Model 1: adjusted for age. Model 2: included all drugs with p-value ≤0.15 in model 1, adjusted for age, Charlson comorbidity index, smoking, body mass index, and number of drugs taken. All models were a bootstrap with resampling of twin pairs for 10,000 times. Bold values are significant medications after adjustments. CI: confidence interval. *medications with p-value p≤0.15 in model 1.

**Supplementary Table 10. Distribution of first assessed study variables for all sample (SATSA, GENDER, and OCTO-Twin), separated by outcome.**

|  | **Functional aging index (n=1,191)** | | | **Cognitive function (n = 1,094)** | | | **Frailty index (n=1,361)** | | |
| --- | --- | --- | --- | --- | --- | --- | --- | --- | --- |
| **Variable** | **SATSA** | **GENDER** | **OCTO-Twin** | **SATSA** | **GENDER** | **OCTO-Twin** | **SATSA** | **GENDER** | **OCTO-Twin** |
| Sex, n (%) |  |  |  |  |  |  |  |  |  |
| Men | 217 (41.1) | 170 (50.4) | 121 (37.1) | 246 (41.6) | 109 (52.2) | 100 (34.0) | 328 (59.3) | 178 (50.6) | 154 (33.8) |
| Women | 311 (58.9) | 167 (49.6) | 205 (62.9) | 345 (58.4) | 100 (47.8) | 194 (66.0) | 225 (40.7) | 174 (49.4) | 302 (66.2) |
| Functional aging index, M (SD) | 47.1 (10.8) | 42.8 (8.7) | 52.5 (10.4) | - | - | - | - | - | - |
| Cognitive function, M (SD) | - | - | - | 56.0 (9.0) | 54.8 (7.0) | 45.1 (9.1) | - | - | - |
| Frailty index, M (SD) | - | - | - | - | - | - | 9.7 (7.0) | 11.5 (6.2) | 20.2 (10.1) |
| Age (years), M (SD) | 65.5 (8.6) | 74.4 (2.7) | 82.8 (2.6) | 63.1 (7.2) | 74.4 (2.6) | 82.8 (2.4) | 65.6 (8.6) | 74.4 (2.7) | 83.2 (2.8) |
| Charlson comorbidity index, M (SD) | 0.14 (0.47) | 0.20 (0.66) | 0.24 (0.63) | 0.11 (0.44) | 0.21 (0.67) | 0.22 (0.59) | 0.16 (0.51) | 0.22 (0.69) | 0.33 (0.76) |
| Body mass index (kg/m^2^), M (SD) | 26.1 (9.1) | 26.6 (3.7) | 24.8 (3.7) | 25.8 (3.9) | 26.6 (3.5) | 24.7 (3.5) | 26.1 (8.9) | 26.6 (3.8) | 24.6 (3.7) |
| Number of drugs, M (SD) | 1.62 (1.52) | 2.61 (2.28) | 2.95 (2.58) | 1.74 (1.74) | 2.50 (2.22) | 3.03 (2.59) | 1.57 (1.43) | 2.66 (2.31) | 3.19 (2.57) |
| Smoke, n (%) |  |  |  |  |  |  |  |  |  |
| Not currently smoking | 445 (84.3) | 305 (90.5) | 303 (92.9) | 479 (81.0) | 194 (92.8) | 277 (94.2) | 464 (83.9) | 320 (90.9) | 416 (91.2) |
| Currently smoking | 83 (15.7) | 32 (9.5) | 23 (7.1) | 112 (19.0) | 15 (7.2) | 17 (5.8) | 89 (16.1) | 32 (9.1) | 40 (8.8) |
| Ace inhibitors, plain (C09A), n (%) |  |  |  |  |  |  |  |  |  |
| No | 516 (97.7) | 336 (99.7) | 326 (100.0) | 584 (98.8) | 208 (99.5) | 294 (100.0) | 538 (97.3) | 352 (100.0) | 456 (100.0) |
| Yes | 12 (2.3) | 1 (0.3) | 0 (0.0) | 7 (1.2) | 1 (0.5) | 0 (0.0) | 15 (2.7) | 0 (0.0) | 0 (0.0) |
| Adrenergics, inhalants (R03A), n (%) |  |  |  |  |  |  |  |  |  |
| No | 522 (98.9) | 322 (95.5) | 312 (95.7) | 574 (97.1) | 197 (94.3) | 282 (95.9) | 548 (99.1) | 337 (95.7) | 436 (95.6) |
| Yes | 6 (1.1) | 15 (4.5) | 14 (4.3) | 17 (2.9) | 12 (5.7) | 12 (4.1) | 5 (0.9) | 15 (4.3) | 20 (4.4) |
| Angiotensin ii receptor blockers (arbs), plain (C09C), n (%) |  |  |  |  |  |  |  |  |  |
| No | 522 (98.9) | 337 (100.0) | 326 (100.0) | 573 (97.0) | 209 (100.0) | 294 (100.0) | 547 (98.9) | 352 (100.0) | 456 (100.0) |
| Yes | 6 (1.1) | 0 (0.0) | 0 (0.0) | 18 (3.0) | 0 (0.0) | 0 (0.0) | 6 (1.1) | 0 (0.0) | 0 (0.0) |
| Antidepressants (N06A), n (%) |  |  |  |  |  |  |  |  |  |
| No | 518 (98.1) | 328 (97.3) | 323 (99.1) | 582 (98.5) | 204 (97.6) | 292 (99.3) | 543 (98.2) | 341 (96.9) | 447 (98.0) |
| Yes | 10 (1.9) | 9 (2.7) | 3 (0.9) | 9 (1.5) | 5 (2.4) | 2 (0.7) | 10 (1.8) | 11 (3.1) | 9 (2.0) |
| Anti-inflammatory and antirheumatic products, non-steroids (M01A), n (%) |  |  |  |  |  |  |  |  |  |
| No | 504 (95.5) | 299 (88.7) | 303 (92.9) | 561 (94.9) | 183 (87.6) | 271 (92.2) | 530 (95.8) | 311 (88.4) | 422 (92.5) |
| Yes | 24 (4.5) | 38 (11.3) | 23 (7.1) | 30 (5.1) | 26 (12.4) | 23 (7.8) | 23 (4.2) | 41 (11.6) | 34 (7.5) |
| Antithrombotic agents (B01A), n (%) |  |  |  |  |  |  |  |  |  |
| No | 496 (93.9) | 281 (83.4) | 313 (96.0) | 566 (95.8) | 179 (85.6) | 285 (96.9) | 521 (94.2) | 291 (82.7) | 439 (96.3) |
| Yes | 32 (6.1) | 56 (16.6) | 13 (4.0) | 25 (4.2) | 30 (14.4) | 9 (3.1) | 32 (5.8) | 61 (17.3) | 17 (3.7) |
| Beta-blocking agents (C07A), n (%) |  |  |  |  |  |  |  |  |  |
| No | 441 (83.5) | 280 (83.1) | 280 (85.9) | 508 (86.0) | 178 (85.2) | 246 (83.7) | 462 (83.5) | 295 (83.8) | 394 (86.4) |
| Yes | 87 (16.5) | 57 (16.9) | 46 (14.1) | 83 (14.0) | 31 (14.8) | 48 (16.3) | 91 (16.5) | 57 (16.2) | 62 (13.6) |
| Blood glucose lowering drugs, excl. Insulins (A10B), n (%) |  |  |  |  |  |  |  |  |  |
| No | 511 (96.8) | 324 (96.1) | 318 (97.5) | 585 (99.0) | 206 (98.6) | 288 (98.0) | 538 (97.3) | 337 (95.7) | 444 (97.4) |
| Yes | 17 (3.2) | 13 (3.9) | 8 (2.5) | 6 (1.0) | 3 (1.4) | 6 (2.0) | 15 (2.7) | 15 (4.3) | 12 (2.6) |
| Calcium (A12A), n (%) |  |  |  |  |  |  |  |  |  |
| No | 520 (98.5) | 331 (98.2) | 318 (97.5) | 583 (98.6) | 204 (97.6) | 287 (97.6) | 546 (98.7) | 346 (98.3) | 446 (97.8) |
| Yes | 8 (1.5) | 6 (1.8) | 8 (2.5) | 8 (1.4) | 5 (2.4) | 7 (2.4) | 7 (1.3) | 6 (1.7) | 10 (2.2) |
| Drugs for constipation (A06A), n (%) |  |  |  |  |  |  |  |  |  |
| No | 522 (98.9) | 329 (97.6) | 312 (95.7) | 580 (98.1) | 205 (98.1) | 281 (95.6) | 548 (99.1) | 340 (96.6) | 428 (93.9) |
| Yes | 6 (1.1) | 8 (2.4) | 14 (4.3) | 11 (1.9) | 4 (1.9) | 13 (4.4) | 5 (0.9) | 12 (3.4) | 28 (6.1) |
| Drugs for peptic ulcer and gastro-oesophageal reflux disease (A02B), n (%) |  |  |  |  |  |  |  |  |  |
| No | 492 (93.2) | 313 (92.9) | 314 (96.3) | 565 (95.6) | 191 (91.4) | 282 (95.9) | 518 (93.7) | 326 (92.6) | 439 (96.3) |
| Yes | 36 (6.8) | 24 (7.1) | 12 (3.7) | 26 (4.4) | 18 (8.6) | 12 (4.1) | 35 (6.3) | 26 (7.4) | 17 (3.7) |
| Emollients and protectives (D02A), n (%) |  |  |  |  |  |  |  |  |  |
| No | 528 (100.0) | 336 (99.7) | 325 (99.7) | 590 (99.8) | 209 (100.0) | 293 (99.7) | 553 (100.0) | 351 (99.7) | 451 (98.9) |
| Yes | 0 (0.0) | 1 (0.3) | 1 (0.3) | 1 (0.2) | 0 (0.0) | 1 (0.3) | 0 (0.0) | 1 (0.3) | 5 (1.1) |
| High-ceiling diuretics (C03C), n (%) |  |  |  |  |  |  |  |  |  |
| No | 510 (96.6) | 319 (94.7) | 281 (86.2) | 575 (97.3) | 201 (96.2) | 254 (86.4) | 530 (95.8) | 329 (93.5) | 380 (83.3) |
| Yes | 18 (3.4) | 18 (5.3) | 45 (13.8) | 16 (2.7) | 8 (3.8) | 40 (13.6) | 23 (4.2) | 23 (6.5) | 76 (16.7) |
| Hypnotics and sedatives (N05C), n (%) |  |  |  |  |  |  |  |  |  |
| No | 510 (96.6) | 316 (93.8) | 262 (80.4) | 555 (93.9) | 201 (96.2) | 233 (79.3) | 534 (96.6) | 330 (93.8) | 365 (80.0) |
| Yes | 18 (3.4) | 21 (6.2) | 64 (19.6) | 36 (6.1) | 8 (3.8) | 61 (20.7) | 19 (3.4) | 22 (6.2) | 91 (20.0) |
| Lipid modifying agent, plain (C10A), n (%) |  |  |  |  |  |  |  |  |  |
| No | 509 (96.4) | 336 (99.7) | 326 (100.0) | 576 (97.5) | 204 (97.6) | 294 (100.0) | 534 (96.6) | 351 (99.7) | 456 (100.0) |
| Yes | 19 (3.6) | 1 (0.3) | 0 (0.0) | 15 (2.5) | 5 (2.4) | 0 (0.0) | 19 (3.4) | 1 (0.3) | 0 (0.0) |
| Opioids (N02A), n (%) |  |  |  |  |  |  |  |  |  |
| No | 509 (96.4) | 325 (96.4) | 304 (93.3) | 571 (96.6) | 200 (95.7) | 273 (92.9) | 534 (96.6) | 340 (96.6) | 427 (93.6) |
| Yes | 19 (3.6) | 12 (3.6) | 22 (6.7) | 20 (3.4) | 9 (4.3) | 21 (7.1) | 19 (3.4) | 12 (3.4) | 29 (6.4) |
| Other analgesics and antipyretics (N02B), n (%) |  |  |  |  |  |  |  |  |  |
| No | 491 (93.0) | 227 (67.4) | 210 (64.4) | 493 (83.4) | 144 (68.9) | 196 (66.7) | 515 (93.1) | 237 (67.3) | 282 (61.8) |
| Yes | 37 (7.0) | 110 (32.6) | 116 (35.6) | 98 (16.6) | 65 (31.1) | 98 (33.3) | 38 (6.9) | 115 (32.7) | 174 (38.2) |
| Selective calcium channel blockers with mainly vascular effects (C08C), n (%) |  |  |  |  |  |  |  |  |  |
| No | 512 (97.0) | 336 (99.7) | 326 (100.0) | 579 (98.0) | 204 (97.6) | 294 (100.0) | 538 (97.3) | 351 (99.7) | 456 (100.0) |
| Yes | 16 (3.0) | 1 (0.3) | 0 (0.0) | 12 (2.0) | 5 (2.4) | 0 (0.0) | 15 (2.7) | 1 (0.3) | 0 (0.0) |
| Thyroid preparations (H03A), n (%) |  |  |  |  |  |  |  |  |  |
| No | 497 (94.1) | 323 (95.8) | 312 (95.7) | 563 (95.3) | 202 (96.7) | 282 (95.9) | 521 (94.2) | 337 (95.7) | 433 (95.0) |
| Yes | 31 (5.9) | 14 (4.2) | 14 (4.3) | 28 (4.7) | 7 (3.3) | 12 (4.1) | 32 (5.8) | 15 (4.3) | 23 (5.0) |
| Urologicas (G04B), n (%) |  |  |  |  |  |  |  |  |  |
| No | 528 (100.0) | 328 (97.3) | 323 (99.1) | 589 (99.7) | 203 (97.1) | 293 (99.7) | 553 (100.0) | 342 (97.2) | 453 (99.3) |
| Yes | 0 (0.0) | 9 (2.7) | 3 (0.9) | 2 (0.3) | 6 (2.9) | 1 (0.3) | 0 (0.0) | 10 (2.8) | 3 (0.7) |
| Vitamin B12 and folic acid (B03B), n (%) |  |  |  |  |  |  |  |  |  |
| No | 518 (98.1) | 319 (94.7) | 302 (92.6) | 587 (99.3) | 195 (93.3) | 273 (92.9) | 542 (98.0) | 333 (94.6) | 423 (92.8) |
| Yes | 10 (1.9) | 18 (5.3) | 24 (7.4) | 4 (0.7) | 14 (6.7) | 21 (7.1) | 11 (2.0) | 19 (5.4) | 33 (7.2) |

Note. M (SD) = mean and standard deviation. n (%) = number of observations and percentage. The level of FI was multiplied by 100 to facilitate interpretation, and the estimates represent increments of 1 in these measures.

**Supplementary Fig. 4. Functional aging index values over age (during all study period, separated by cohort).**

**
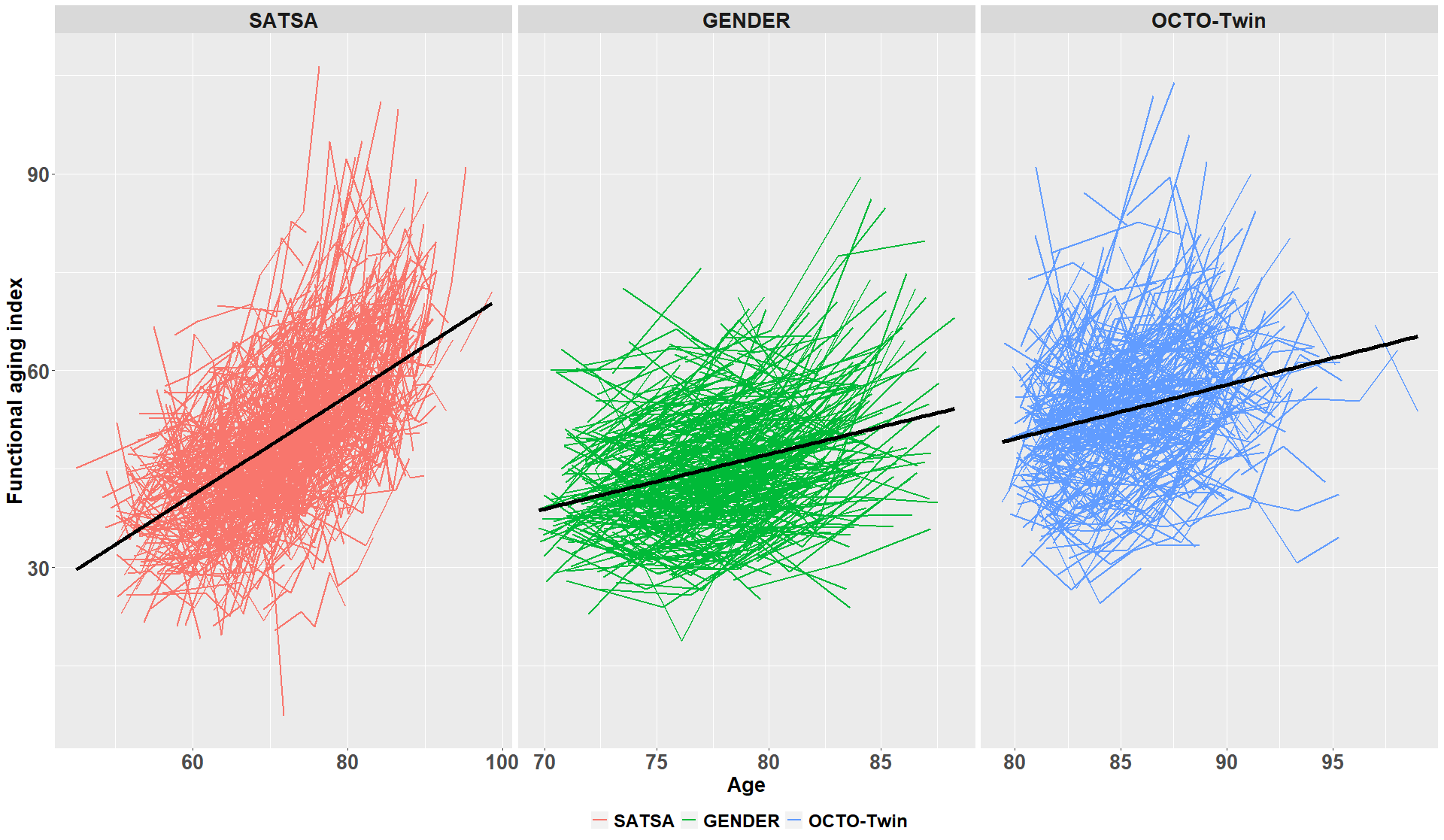
**

Note. The functional aging index (FAI, n= 1,191) has four domains: sensory functioning (hearing and vision), lung function, grip strength (corrected for sex), and gait. A higher score means worse performance/less ability. Each line in all graphs represents the participant’s measurements over age in different IPTs. The black line represents a smoothing trend-line considering linear model as a method. SATSA participants’ age varies between 45 and 99 years. For GENDER, it varies between 69 and 88 years. Lastly, for OCTO-Twin, it varies between 79 and 100 years. For FAI and FI, the graphs show increasing values of the indexes over age. The COG graph shows decreasing values of cognitive function over age.

**Supplementary Fig. 5. Cognitive function values over age (during all study period, separated by cohort).**

**
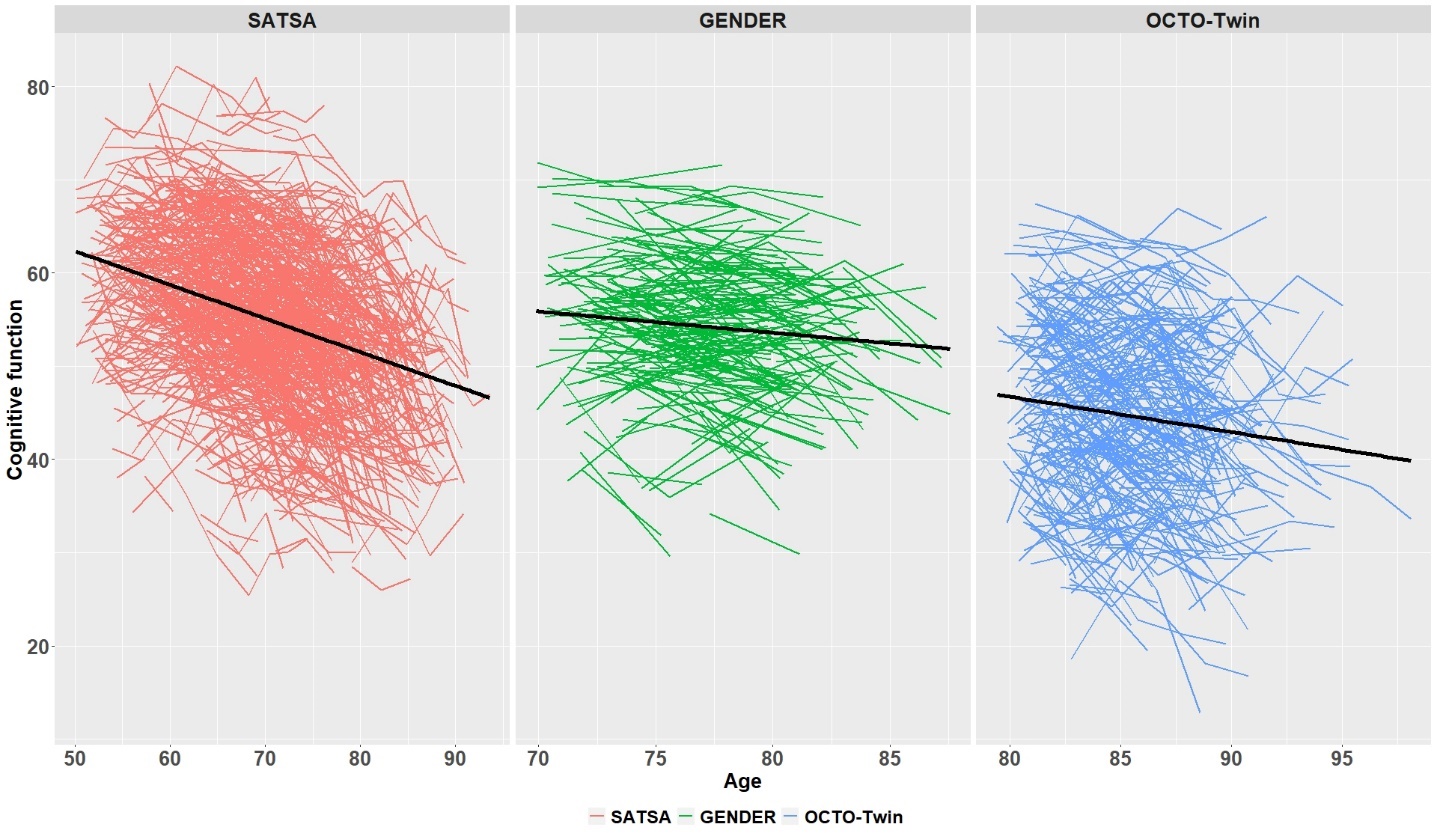
**

Note. The cognitive function (COG, n= 1,094) consists of a general cognitive ability measure (score) composed of distinct types of cognitive tests covering processing speed, verbal and spatial abilities, and memory (episodic and working memory). Higher values, better cognitive function. Each line in all graphs represents the participant’s measurements over age in different IPTs. The black line represents a smoothing trend-line considering linear model as a method. SATSA participants’ age varies between 45 and 99 years. For GENDER, it varies between 69 and 88 years. Lastly, for OCTO-Twin, it varies between 79 and 100 years. For FAI and FI, the graphs show increasing values of the indexes over age. The COG graph shows decreasing values of cognitive function over age.

**Supplementary Fig. 6.** **Frailty index values over age (during all study period, separated by cohort).**

**
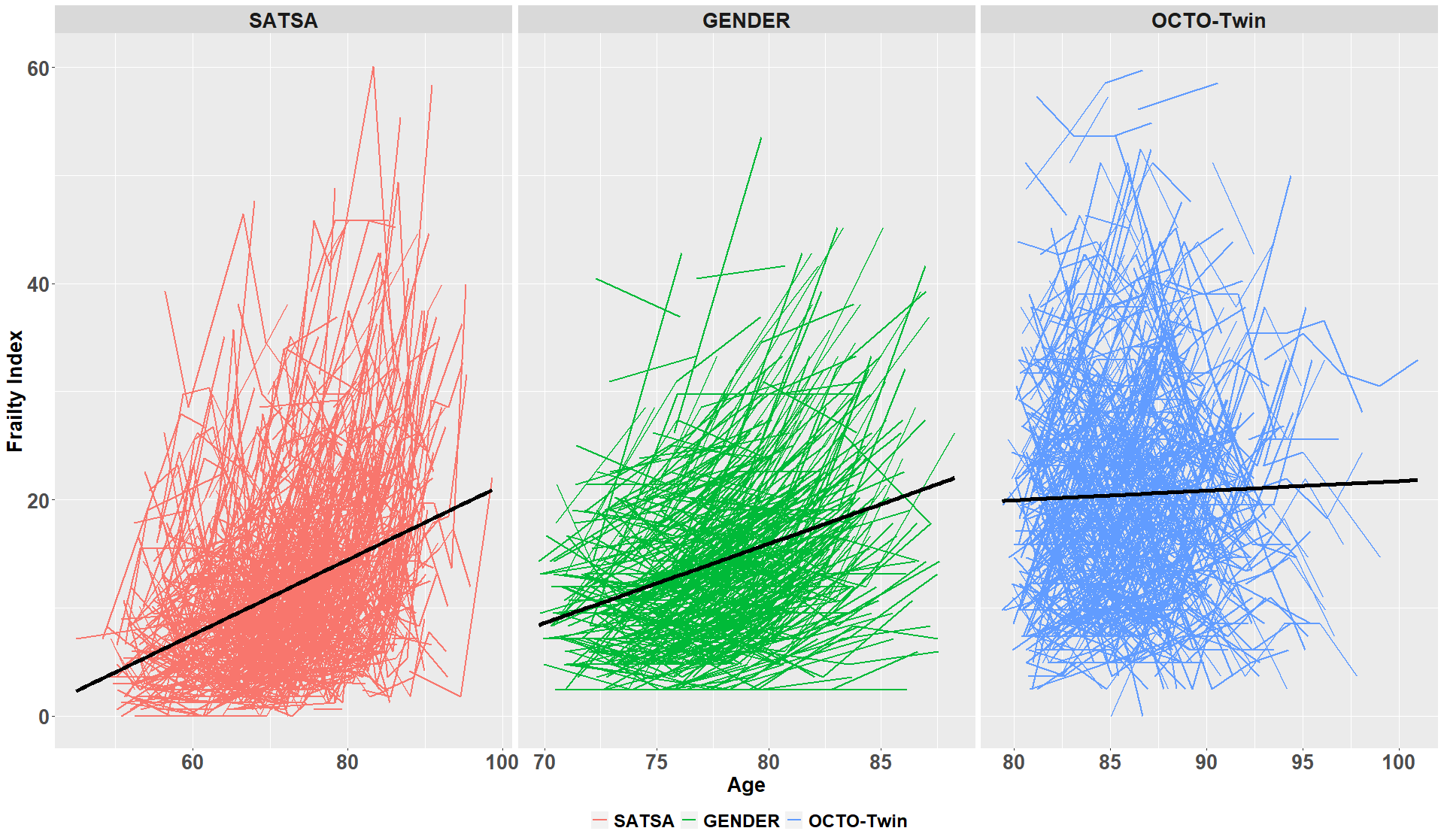
**

Note. The frailty index (FI, n= 1,361) was based on the accumulation of deficits approach. The index ranges from 0 to 1.0, with values closer to one representing more frailty. The level of FI was multiplied by 100 to facilitate interpretation, and the estimates represent increments of 1 in these measures. Each line in all graphs represents the participant’s measurements over age in different IPTs. The black line represents a smoothing trend-line considering linear model as a method. SATSA participants’ age varies between 45 and 99 years. For GENDER, it varies between 69 and 88 years. Lastly, for OCTO-Twin, it varies between 79 and 100 years. For FAI and FI, the graphs show increasing values of the indexes over age. The COG graph shows decreasing values of cognitive function over age.

**Supplementary Table 11. Years of follow up and number of IPTs for functional aging index, cognitive function, and frailty index samples.**

|  |  | **Functional Aging index (n=1,191)** | **Cognitive function (n=1,094)** | **Frailty index (n=1,361)** |
| --- | --- | --- | --- | --- |
| Years of follow-up | Minimum | 1.8 | 1.8 | 1.8 |
|  | Mean | 8.7 | 9.5 | 8.7 |
|  | Maximum | 24.4 | 26.9 | 24.4 |
| Number of IPTs | Minimum | 1 | 1 | 1 |
|  | Median | 2 | 2 | 2 |
|  | Maximum | 8 | 9 | 8 |

**REFERENCES**

Finkel, D., Sternäng, O., Jylhävä, J., Bai, G., & Pedersen, N. L. (2019). Functional Aging Index Complements Frailty in Prediction of Entry Into Care and Mortality. *The Journals of Gerontology. Series A, Biological Sciences and Medical Sciences*, *74*(12), 1980–1986. https://doi.org/10.1093/GERONA/GLZ155
